# Catalytic Mechanism of SARS-CoV-2 3-Chymotrypsin-Like Protease as Determined by Steady-State and Pre-Steady-State Kinetics

**DOI:** 10.1021/acscatal.4c04695

**Published:** 2024-11-27

**Authors:** Jiyun Zhu, Alexandria M. Kemp, Bala C. Chenna, Vivek Kumar, Andrew Rademacher, Sangho Yun, Arthur Laganowsky, Thomas D. Meek

**Affiliations:** † Departments of Biochemistry and Biophysics, Texas A&M University, College Station, Texas 77843, United States; ‡ Chemistry, Texas A&M University, College Station, Texas 77843, United States

**Keywords:** 3CL protease, Main protease, pH-rate profiles, solvent kinetic isotope effects, COVID-19, SARS-CoV-2

## Abstract

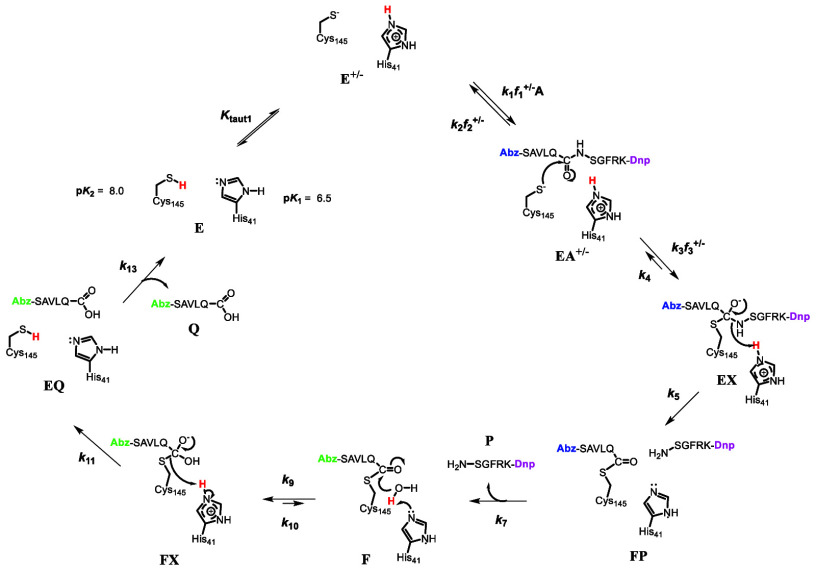

The 3-chymotrypsin-like protease (3CL-PR; also known as Main protease) of SARS-CoV-2 is a cysteine protease that is the target of the COVID-19 drug, Paxlovid. Here, we report for 3CL-PR, the pH-rate profiles of a substrate, an inhibitor, affinity agents, and solvent kinetic isotope effects (sKIEs) obtained under both steady-state and pre-steady-state conditions. “Bell-shaped” plots of log(*k*_cat_/*K*_a_) vs pH for the substrate (Abz)SAVLQ*SGFRK(Dnp)-NH_2_ and p*K*_i_ vs pH for a peptide aldehyde inhibitor demonstrated that essential acidic and basic groups of p*K*_2_ = 8.2 ± 0.4 and p*K*_1_ = 6.2 ± 0.3, respectively, are required for catalysis, and the pH-dependence of inactivation of 3CL-PR by iodoacetamide and diethylpyrocarbonate identified enzymatic groups of p*K*_2_ = 7.8 ± 0.1 and p*K*_1_ = 6.05 ± 0.07, which must be unprotonated for maximal inactivation. These data are most consistent with the presence of a neutral catalytic dyad (Cys-SH-His) in the 3CL-PR free enzyme, with respective p*K* values for the cysteine and histidine groups of p*K*_2_ = 8.0 and p*K*_1_ = 6.5. The steady-state sKIEs were ^D_2_O^(*k*_cat_/*K*_a_) = 0.56 ± 0.05 and ^D_2_O^*k*_cat_ = 1.0 ± 0.1, and sKIEs indicated that the Cys-S^–^-HisH^+^ tautomer was enriched in D_2_O. Presteady-state kinetic analysis of (Abz)SAVLQ*SGFRK(Dnp)-NH_2_ exhibited transient lags preceding steady-state rates, which were considerably faster in D_2_O than in H_2_O. The transient rates encompass steps that include substrate binding and acylation, and are faster in D_2_O wherein the more active Cys-S^–^-HisH^+^ tautomer predominates. A full catalytic mechanism for 3CL-PR is proposed.

## Introduction

The COVID-19 pandemic, caused by the severe acute respiratory syndrome coronavirus-2 (SARS-CoV-2).^1^ emerged from Wuhan, China in 2019, and has resulted in global totals of 761 million cases and 6.8 million deaths as of September 2023.^2^ Historically, COVID-19 now ranks fifth among the deadliest pandemics.^3^ While the availability of vaccines since the end of 2020 has significantly contributed to the control of COVID-19, to the extent that the World Health Organization declared an end to its public emergency status in May 2023, new strains of SARS-CoV-2 (variants of concern) continue to emerge,^4^ requiring updating to expand the efficacy of existing vaccines. Worldwide, cases of COVID-19 are again on the rise as of late 2023 during which time hospitalizations and deaths from COVID-19 have increased nearly 8% in the United States.^5^ A single effective therapy for COVID-19, Paxlovid, has been approved by the FDA for emergency use.^6−9^ Its active component, nirmatrelvir, is a reversible covalent inhibitor of the cysteine protease SARS-CoV-2 3-chymotrypsin-like protease^8,9^ (3CL-PR; also known as Main protease, Uniprot number P0CTC1, E.C. 3.4.22.69).^10,11^ 3CL-PR, a homodimer of 34 kDa subunits, catalyzes 11 peptide cleavages of coronaviral polyproteins to initiate maturation of the nonstructural proteins and the essential enzymes of the virus.^14,15^ Paxlovid comprises an important milestone in drug discovery: (1) it unequivocally validates 3CL-PR as a drug target for COVID-19 and (2) it is the first of myriad inhibitors^12,13^ of cysteine proteases to become a marketed drug. Recently, new strains of SARS-CoV-2 contain mutations within the substrate-binding site of 3CL-PR, which result in diminished inhibition by nirmatrelvir (Paxlovid), suggesting that it will ultimately become ineffective.^16,17^ Accordingly, the discovery of second-generation drug-quality inhibitors of SARS-CoV-2 3CL-PR remains a necessity.

Detailed kinetic studies of the catalytic mechanisms of cysteine proteases including SARS-CoV 3CL-PR^18,19^ papain,^20−23^ cruzain,^24^ and cathepsin C^25^ all conform to the generic double-displacement mechanism, which comprises two half-reactions: *S*-acylation of the active-site cysteine by the scissile peptide, followed by deacylation, or hydrolysis of the *S*-acyl enzyme. The protonation state of the Cys–His catalytic dyad in the free enzyme or the enzyme–substrate complex is either the neutral thiol-imidazole form or the imidazolium-thiolate form, and the “tautomerization”^20−22,24,25^ between these protonation states has been the subject of considerable debate.^26−28^ Kinetic data for papain,^20−23^ human cathepsin C,^24^ and SARS-CoV 3CL-PR^18,19^ are consistent with an Cys-S^–^-H-His^+^ ion pair in the free enzyme, while for cruzain this dyad is neutral.^24^ However, molecular modeling studies have indicated that the catalytic dyad of SARS-CoV-2 3CL-PR is neutral (Cys-SH---His) for both the free enzyme^29,30^ and an enzyme–inhibitor complex.^31^ For those inhibitors that form covalent bonds with the active-site cysteine via electrophilic “warheads”,^18,19^ knowledge of the protonation states of the two catalytic residues of the protease comprises important information for the rational design of potential covalent inhibitors because the thiol form of the cysteine residue is far less reactive than the thiolate.^32^ Furthermore, understanding of which catalytic steps in an enzymatic mechanism are rate-limiting will also assist in the rational design of inhibitors since blockade of the slowest steps constitutes the most efficient path to inhibition. Here, we employ solvent kinetic isotope effects (sKIEs) and pH-rate profiles of a substrate, an inhibitor, and affinity agents to investigate the protonation state(s) of 3CL-PR, and pre-steady-state kinetics to ascertain the rate-limiting steps of its catalytic mechanism.

## Materials and Methods

### Chemicals and Molecular Biology Reagents

Chemical reagents for protein purification, chemical synthesis, and enzyme assays were obtained from commercial suppliers and used without further purification, unless otherwise stated. Genes and plasmids used for the expression of 3CL-PR were prepared by Genscript and any subsequent subcloning of these genes was verified by sequencing. The FRET-based peptide substrate *N*^α^-(Dabcyl)-KTSAVLQ*SGFRKME(Edans) (trifluoroacetate salt) was purchased from BPS Bioscience (Cat. no. 79953, San Diego, CA) and used without further purification. Stock solutions were prepared in 100% DMSO.

The preparation and characterization of wild-type and the Cys_145_Ala mutant of SARS-CoV-2 3CL-PR, the FRET-based peptide substrate (Abz)-SAVLQ*SGFRK(Dnp), and the peptide aldehyde inhibitor BC-666 are described in the Supporting Information.

Evaluation of a second FRET-based substrate for 3CL-PR was provided by (Dabcyl)KTSAVLQ*SGFRKME(Edans). We measured the fluorescence of the amino product H_2_N-SGFRKME(Edans) to ascertain the excitation and emission wavelengths to be used for the measurement of fluorescence as well as the background fluorescence of the intact peptide. Values of λ_ex_ = 342 nm and λ_em_ = 520 nm provided optimal conditions for the evaluation of 3CL-PR-catalyzed peptidolysis of this substrate. We developed standard curves to quantify fluorescent product H_2_N-SGFRKME(Edans)-NH_2_. The steady-state kinetics of the 3CL-PR-catalyzed peptidolysis of (Dabcyl)KTSAVLQ*SGFRKME(Edans)-NH_2_ were conducted to compare the *k*_cat_ and *k*_cat_/*K*_m_ values with those of (Abz)SAVLQ*SGFRK(Dnp).

## pH-Rate Profiles

### Stability of 3CL-PR at Variable pH

Mixed buffers at 5× concentrations were prepared (250 mM sodium acetate, 250 mM MES, 500 mM TEA, 750 mM NaCl and 5 mM Na_2_EDTA, for values of pH = 5.0–6.0 at 0.25 increments) and for pH = 6.25–9.0 (500 mM DEA, 250 mM MES, 250 mM TAPSO, 750 mM NaCl and 5 mM Na_2_EDTA). These mixed buffers provide constant ionic strengths.^33^ Reaction mixtures were prepared from solutions of these 5× mixed buffers, 100 mM DTT, 50 mg/mL BSA, and 20 μM 3CL-PR, which contained final concentrations of 1× mixed buffer (at all values of pH), 2 mM DTT, 5 mg/mL BSA, and 5 μM 3CL-PR CL-PR, and these mixtures were then incubated on ice for 1 h. 2 μL enzyme aliquots at each value of pH was diluted 25-fold into a mixture containing 1× mixed buffer (pH 7.5), 2 mM DTT, 0.5 mg/mL BSA in order to neutralize the preincubation mixtures. Residual 3CL-PR activities were measured upon the addition of diluted enzyme stock solutions into master mix solutions containing 40 μM (final) (Abz)SAVLQ*SGFRK(Dnp) in a BioTek 96-well plate reader at λ_ex_/λ_em_ = 320/420 nm for 20 min. Initial velocities in units of RFU/min were ascertained, and residual initial velocities at all pH values were compared to that at pH = 7.5 to determine the stability of 3CL-PR over the germane range of values of pH used in our study.

We acquired initial velocity data for the 3CL-PR-catalyzed peptidolysis of (Abz)SAVLQSGFRK(Dnp)-NH_2_ at pH values of 5.0–9.0. For reaction mixtures obtained in H_2_O, glycerol was added to a final concentration of 9% (v/v) in order to obtain the same solution viscosity as data obtained in samples prepared in ∼100% deuterium oxide.^34^ Reaction mixtures (100 μL) contained final concentrations of 1× mixed buffer (50 mM sodium acetate, 50 mM MES, 50 mM TEA, 150 mM NaCl and 1 mM Na_2_EDTA, for pH = 5.0–6.0 at 0.25 increments, and for pH = 6.25–9.0, (100 mM DEA, 50 mM MES, 50 mM TAPSO, 150 mM NaCl and 1 mM Na_2_EDTA),^24^ with all solutions containing 2 mM DTT, 9% (v/v) glycerol, 10% (v/v) DMSO and 0.0–150 μM substrate in Greiner 96-well plates. Reactions were initiated by the addition of 10 μL of 200 nM 3CL-PR (in 1× buffer, 2 mM DTT and 0.5 mg/mL BSA, to a final concentration of 20 nM 3CL-PR), and 20 min time courses of increasing fluorescence were acquired using a BioTek SynergyMx plate reader (Agilent Technologies, Santa Clara, CA) at λ_ex_ = 320 nm and λ_em_ = 420 nm. Initial velocities (RFU/min) were exported from the Gen5.0 software and converted to units of μM/s by use of a calibration curve in which the RFU values of the fluorescent product H_2_N-SGFRK(Dnp)-NH_2_ were plotted vs their micromolar concentrations.

pH-dependence of inhibition of 3CL-PR by a peptide aldehyde inhibitor (BC-666). 4-Methyl-*N*-((*S*)-1-oxo-1-(((*S*)-1-oxo-3-(2-oxo-1,2-dihydropyridin-3-yl)propan-2-yl)amino)-3-phenylpropan-2-yl)piperazine-1-carboxamide (BC-666, NMePip-Phe-2Pyrd-CHO) is a peptidomimetic aldehyde which is a competitive inhibitor of 3CL-PR (*K*_*i*_ = 600 ± 100 nM) (Figure S16). The inhibition constant of BC-666 was measured at pH 5.0–9.0 at a single fixed concentration (30 μM) of the substrate (Abz)SAVLQSGFRK(Dnp)-NH_2_. In Greiner 96-well plates (black, clear bottom, half-area), into each well was added 80 μL of a reaction mixture made from 5× mixed buffers, 50 mM TCEP or 100 mM DTT, and water. Then, 5 μL of 600 μM of (Abz)SAVLQSGFRK(Dnp)-NH_2_ substrate stock solution in 100% DMSO was added into the mixtures, followed by the addition of another 5 μL of a 20× stock solution of inhibitor (BC-666) (0–200 μM) in 100% DMSO. After thorough mixing, 10 μL of a stock solution of 200 nM 3CL-PR (in 1× buffer, 1 mM TCEP, 0.1% (v/v) Triton X-100) was added to reaction mixtures containing the substrate and inhibitor. Final concentrations in each plate well were as follows: 1× mixed buffer (pH 5.0–9.0), 1 mM TCEP (when inhibitors or affinity agents were studied), or 2 mM DTT, 10% (v/v) DMSO, 30 μM substrate, and 20 nM 3CL-PR (all final concentrations). The fluorescence intensity of each well was monitored using BioTek SynergyMx plate reader at λ_ex_/λ_em_ = 320/420 nm for at least 20 min, and these data were converted into initial velocities.

### Solvent-Kinetic Isotope Effects

Solvent kinetic isotope effects (SKIEs) were evaluated from the pH- and pD-dependence of the kinetic parameters *k*_cat_ and *k*_cat_/*K*_a_. Identical 5× mixed buffers and 5× reaction mixtures (1× containing 2 mM DTT and 5 mg/mL BSA) as those used in the pH-rate profiles were prepared in 100% D_2_O and adjusted to the desired value of pD using either sodium deuteroxide or deuterium chloride (pD 5.0–9.0). The calculated final percentage of deuterium oxide in these buffers was 93%, based on precedent,^25^ and pD values were determined as the measured pH value +0.4.^35^ sKIEs were then ascertained from the comparison of the pL-independent values of these kinetic parameters.^35^ Glycerol was not added to the mixed buffers prepared in D_2_O. Initial velocity data were acquired identically in both solvents at corresponding values of pL = 5.0–9.0. Additional samples were prepared in H_2_O, which contained 9% (v/v) glycerol in order to complement the solution viscosity of the buffers made in D_2_O.

### pH-Dependence of Inactivation of 3CL-PR by Iodoacetamide

The time-dependent kinetics of inactivation by 0–1 mM concentrations of the affinity label iodoacetamide (IAM) were conducted at pH values ranging from 6.0 to 9.0 using the Mixed Buffers described above. At each pH value, 2 μM of 3CL-PR was preincubated with 0–1 mM IAM in 1× buffer [diluted from 5× mixed buffers (pH = 6.0–9.0) containing 0.5 mg/mL BSA (DTT is omitted from the assay solution to prevent side reaction from IAM)] on ice for 10–60 min. Aliquots of 1 μL of the preincubation mixtures were then diluted 100-fold for each time point into reaction mixtures (25 °C) containing 1× mixed buffer (pH = 6.0–9.0), 30 μM Abz-SAVLQSGFRK(Dnp)-NH_2_, 2 mM DTT, 0.5 mg/mL BSA, and 10% DMSO to quench the inactivation reaction. Residual initial velocity data were collected on a plate reader as above, and at each value of pH, residual activity of 3CL-PR  at each time point and concentration of inactivator, were acquired for which *v*_0_ and *v*_i_ are the residual initial velocities measured in the respective absence and presence of IAM.

### pH-Dependence of Affinity Labeling with Diethylpyrocarbonate

The time-dependent kinetics of inactivation by 0–1 mM concentrations of the affinity label diethylpyrocarbonate (DEPC) were conducted at pH = 5.5–9.0, with slight modifications of experimental conditions to accommodate the inherent hydrolytic instability of DEPC (prepared freshly as solutions in 100% (v/v) ethanol and stored on ice). For each pH value, 2 μM of 3CL-PR was preincubated with 0–10 mM DEPC in 1× buffer (diluted from 5× mixed buffers), 1 mM TCEP, 0.01% (v/v) Triton X-100, 10% (v/v) ethanol (from DEPC stock solutions) on ice for 10–60 s, and then 1 μL aliquots were withdrawn and diluted 100-fold into reaction mixtures containing 1× mixed buffer (pH = 5.5–9.0), 30 μM Abz-SAVLQSGFRK(Dnp)-NH_2_ substrate, 1 mM TCEP, 0.01% (v/v) Triton X-100, 10% v/v DMSO to quench the inactivation reaction. Residual initial velocity data were collected on a plate reader, for which *v*_0_ and *v*_i_ are as described above.

### Native Mass Spectrometry

Proteins for native mass spectrometry (MS) studies were prepared as previously reported.^36^ In brief, samples of both wild-type and the C145A mutant of 3CL-PR were diluted to 5 μM, and the buffer was exchanged into 200 mM ammonium acetate (pH 7.0) with a spin-dialysis column (Micro Bio-Spin 6, Bio-Rad). For a sample containing a 5-fold stoichiometric excess of BC-666, a 25 μM final concentration was added to the sample prior to the buffer exchange, followed by incubation at 25 °C for 20 min, and the excess unbound BC-666 was removed using a spin-dialysis column (Micro Bio-Spin 6, Bio-Rad). Upon buffer exchange, samples were diluted to 1.25 μM protein with 200 mM ammonium acetate for native MS analysis. Wild-type 3CL-PR (5 μM) was pretreated with a 40-fold molar excess of iodoacetamide in the presence or absence of BC-666 (25 μM final concentration), and the sample was incubated at 25 °C for 20 min prior to buffer exchange and analysis by native MS. In a second sample, BC-666 was removed by buffer exchange after 20 min of incubation at 25 °C, and a 40-fold molar excess of iodoacetamide was added to the buffer-exchanged sample. The sample was incubated for 20 min at 25 °C prior to native MS analysis. In an additional study, we treated buffer-exchanged wild-type 3CL-PR (5 μM) with 10 μM DEPC, and analyzed the products by native MS. Native MS data were collected on a Thermo Exactive Plus instrument with an Ultra High Mass Range Orbitrap mass spectrometer (Thermo Scientific) with source DC offset at 21.0 V, Injection flatapole DC at 20.0 V, inter flatapole lens at 9.0 V, bent flatapole DC at 3.0 V, trapping gas pressure at 5.0, in-source CID at 20.0 eV, CE at 30.0 V, Desolvation voltage at −25.0 V, spray voltage at 1.4 kV, capillary temperature at 250 °C, scanning from 500 to 12,000 *m*/*z* at resolution 12,500. Native MS data were deconvoluted using UniDec.^37^

### Pre-steady-State Kinetics Using Stopped-Flow Fluorimetry of Two FRET-Based Substrates

Pre-steady-state kinetics of the 3CL-PR-catalyzed peptidolysis of the Abz-SAVLQSGFRK(Dnp)-NH_2_ substrate were conducted at 25 °C and at pH(D) = 7.5. Time courses of the fluorescence of the product Abz-SAVLQ-COOH were acquired at 0.002–0.25 s using a KinTek Auto-SF stopped-flow fluorimeter apparatus (Kintek Corporation, Snow Shoe, PA). Total reaction volumes of 60 μL were obtained upon rapid mixing of 30 μL solutions in either H_2_O or 93% D_2_O containing 2× final concentrations of enzyme (1× = 10 μM) (Syringe A), 100 mM DEA, 50 mM MES, 50 mM TAPSO (pL = 7.5), 150 mM NaCl, 1 mM Na_2_EDTA, 10% (v/v) DMSO, 2 mM TCEP, with 30 μL solutions in either H_2_O or 93% D_2_O (Syringe B) of 2× concentrations of Abz-SAVLQSGFRK(Dnp)-NH_2_ (1× = 20–120 μM) in buffers containing 100 mM DEA, 50 mM MES, 50 mM TAPSO (pL = 7.5), 150 mM NaCl, 1 mM Na_2_EDTA, 10% (v/v) DMSO, and 2 mM TCEP. To ensure that the overall relative fluorescence of product formation did not exceed the instrumental limit of detection, the substrate stock solution with the highest concentration was injected with the enzyme stock solution to achieve the full hydrolysis to acquire the highest amount of fluorescent product, then the photomultiplier tube voltage and the light source intensity were adjusted to ensure that the resulting relative fluorescence was stable below but close to 10 RFUs). The instrument setting in the final reaction was 50% of the light source sensitivity at 300 mA, with an excitation wavelength of 320 nm and fluorescence measured at 420 nm. Two thousand data points were collected for 120 s in the form of time and log(time) (for calibration), and the first 0.25 s were evaluated. Each set of experiments was repeated at least 8 times, and the final data points used for calculation were the averaged data from the 8 replicates. The experiment was then repeated in buffers prepared in D_2_O, with the same procedure applied. Relative fluorescence data were converted to [product] (μM) using the standard curve acquired with concentrations of the fully hydrolyzed substrates, and averaged data of [product] vs time at each concentration of substrates were collected for analysis. Likewise, pre-steady-state kinetic data for the 3CL-PR-catalyzed hydrolysis of (Dabcyl)KTSAVLQ*SGFRKME(Edans)-NH_2_ (5–90 μM) in both H_2_O and D_2_O were generated at pH(D) = 7.5 in a stopped-flow fluorimeter, by measurement of fluorescence at 520 nm at 2–200 ms. Final concentrations of 3CL-PR were 10 μM in these studies. In a second series of pre-steady-state measurements, the fluorescence produced by rapid mixing of (Dabcyl)KTSAVLQ*SGFRKME(Edans)-NH2 (5, 30, and 60 μM; final concentrations) was measured in H_2_O-containing buffer with final concentrations of 10 μM of wild-type or C145A 3CL-PR.

### Data Analysis

Initial rates (μM/s) were plotted vs μM concentrations of substrate and fitted to the Michaelis–Menten equation (eq 1) in which *v* is the initial rate at each substrate concentration (*A*), *V*_max_ and
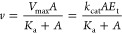
1*k*_cat_ are, respectively, the maximum velocity and turnover number, *E*_t_ is the concentration of 3CL-PR, *A* is the concentration of substrate, and *K*_a_ is the Michaelis constant of the substrate.

Inhibition data conforming to apparent competitive, noncompetitive (mixed), or uncompetitive inhibition were fitted to eqs 2–4, in which *v* is the initial rate, *V*_max_ is the apparent maximum velocity, *A* is the variable substrate concentration, *K*_a_ is the apparent Michaelis constant, *I* is the concentration of inhibitor, and *K*_is_ and *K*_ii_ are the respective slope and intercept inhibition constants.
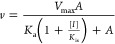
2
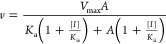
3
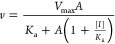
4pH-rate profiles corresponding to bell-shaped curves in which apparent slopes of 1 and −1 are observed at low and high pH were fitted to eq 5 in which y is the experimental value of *k*_cat_/*K*_a_, *c* is the pH- or pD-independent values of the kinetic parameter, and p*K*_1_ and p*K*_2_ are the apparent acid and base dissociation constants of groups on either the enzyme or substrate.
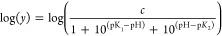
5

For pH- and pD-rate profiles of log(*k*_cat_) vs (pL = pH or pD) that exhibited an apparent slope of 1 at low pL while at high pL leveled off to a lower value of log(*k*_cat_) (a “half-bell-wave” profile), data were fitted to eq 6 in which y is *k*_cat_ or a kinetic parameter of inactivation (*k*_inact_/*K*_I_), *Y*_L_ and *Y*_H_ are pL-independent values of *k*_cat_ at low and high values of pL, respectively, and p*K*_1_ and p*K*_2_ are the respective acid and base dissociation constants.
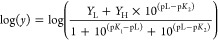
6

The theory leading to derivations of eqs 1–6 may be found in refs (38–40). Time-dependent inhibition or inactivation data were fitted to the Cheng–Prusoff^41^ equation (eq 7), in which *v*_i_ and *v*_0_ is the respective initial velocity in the presence or absence of an inhibitor or inactivator, *K*_a_ is the Michaelis constant acquired at each value of pH, [*I*] is the inhibitor concentration, *K*_i_ is the inhibition constant (*K*_I_ in the case of an inactivator), and [*A*] is the substrate concentration in the assay.
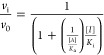
7

Data plotted as 1/log(*K*_i_) vs pH, which exhibited a bell-shaped curve with apparent slopes of 1 (low pH) and −1 (high pH), were fitted to eq 5, in which *y* is the experimental value of p*K*_i_, *c* is the pH-independent value *K*_i_, and p*K*_1_ and p*K*_2_ are the respective acid and base dissociation constants.

For inhibitors and affinity labeling agents that exhibited time-dependent inhibition or inactivation of 3CL-PR, time courses of product fluorescence (RFUs) were fitted to eq 8, in which *P* is the product concentration (μM), *t* is time in minutes or seconds, *v*_i_ and *v*_s_ are the respective initial and steady-state rates, *C* is a constant due to background fluorescence from the substrate, and *k*_obs_ is the inhibitor- or inactivator-dependent rate of conversion of *v*_i_ to *v*_s_.^41,42^
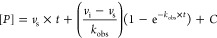
8

**Scheme 1:**
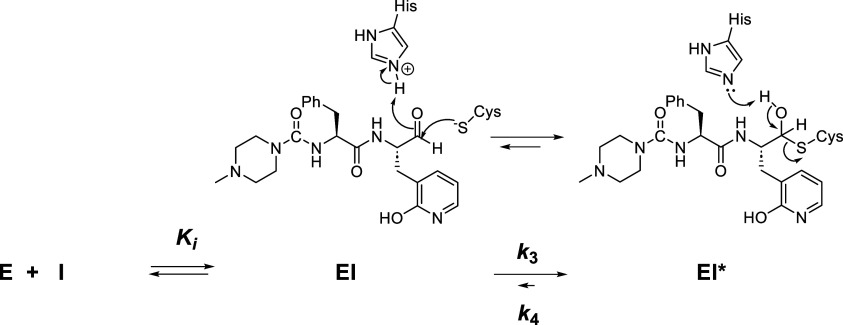


Values of *k*_obs_ were then replotted vs the concentration of inhibitor or inactivator (*I*) using eq 9, in which *k*_3_ and *k*_4_ are the respective rate constants of the formation and reversal of the reversible (inhibitor) or irreversible (*k*_4_ = 0 for an inactivator, see Scheme 1) complex EI*, and *K*_I_ is the inhibition constant describing formation of the initial EI complex.
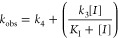
9

The sKIE of the inactivation of 3CL-PR by an affinity label was calculated by dividing the value of *k*_3_ obtained in H_2_O by the value of *k*_3_ obtained in D_2_O.

### pH-Dependence of Inactivation of 3CL-PR by Iodoacetamide

Residual initial velocity data of 3CL-PR treated with affinity-labeling agents  were fitted to eq 10,^38^ for which *v*_0_ and *v*_i_ are the residual initial velocities measured in the respective absence and presence of inactivator, *k*_obs_ is the apparent rate constant of inactivation wherein *k*_obs_ = (*k*_inact_/*K*_I_)[*I*], in which *k*_inact_/*K*_I_ is the second-order rate constant of inactivation, *I* is the concentration of the affinity label, and *t* is the preincubation time in minutes.
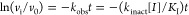
10

Plots of the calculated values of *k*_inact_/*K*_I_ vs pH for which a “half-bell”-shaped plot was observed (*k*_inact_/*K*_I_ decreased with a slope of 1 as pH decreases) were fitted to eq 11 for which c is the pH-independent value of *k*_inact_/*K*_I_ and p*K*_1_ is an acid dissociation constant.
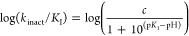
11

For stopped-flow fluorometric data in which relative fluorescence data (RFUs) were collected from 0 to 200 ms, time courses were fitted to eq 12, in which [*P*] is the concentration of product (μM), *t* is seconds, *k*_ss_ is the apparent steady-state rate, β is the amplitude of the lag phase, and λ is the transient rate, and expressions for these three kinetic parameters which are dependent on the substrate concentrations ([*A*]) are respectively given by eqs 13–15, for which the turnover number is *k*_cat_ (s^–1^), [*E*_t_] is [3CL-PR], *K*_a_ and *K*_ia_ are the respective Michaelis and dissociation constants of substrate, and *k*_3_ and *k*_5_ are the individual rate constants for the acylation and deacylation steps, respectively, which represent the respective enzyme-bound formation of and desorption of the fluorescent product Abz-SAVLQ-COOH. The theory behind the derivations eqs 12–16 may be found in ref (40).
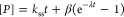
12
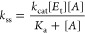
13
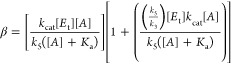
14
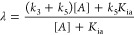
15

In addition, data were fitted globally to eq 16, in which [*F*] refers to the concentration of acyl-3CL-PR, and [*Q*] is the concentration of the fluorogenic product Abz-SAVLQ-COOH.
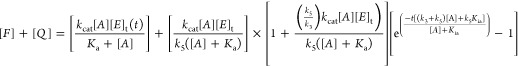
16

Data for pre-steady-state burst kinetics for the substrate (Dabcyl)-KTSAVLQ*SGFRKME-(Edans)-NH_2_ in which the fluorescent product H-SGFRKME(Edans)-NH_2_ is formed were fitted to eq 17, in which in which [*P*] refers to the micromolar concentrations of the aggregate fluorescence observed for the enzyme-bound, unreacted substrate and the product H-SGFRKME-(Edans)-NH_2_, *k*_cat_ is the turnover number (s^–1^), [*E*_t_] is [3CL-PR], *K*_a_ and *K*_ia_ are the respective Michaelis and dissociation constants of substrate.
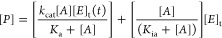
17

Data were fitted and plotted using either Prism (Graph Pad Software, Inc.) or SigmaPlot (Systat Software, Inc.).

## Results and Discussion

### Steady-State Kinetics of FRET-Based Substrates

Michaelis–Menten kinetics of two FRET-based substrates of 3CL-PR, (Abz)SAVLQSGFRK(Dnp)-NH_2_ and (Dabcyl)KTSAVLQSGFRKME(Edans)-NH_2_ (pH 7.5) (Figure S15), provided the following kinetic parameters: *k*_cat_ = 1.02 ± 0.08 s^–1^ and *K*_a_ = 43 ± 9 μM for (Abz)SAVLQSGFRK(Dnp)-NH_2_ and for (Dabcyl)KTSAVLQSGFRKME(Edans)-NH_2_, *k*_cat_ = 0.39 ± 0.01 s^–1^ and *K*_a_ = 38 ± 4 μM. While the Michaelis constants of the two substrates were equivalent, the *k*_cat_ of the longer peptide substrate, which contains more hydrophobic fluorescent groups than (Abz)SAVLQSGFRK(Dnp)-NH_2_, is 39% lower than that of the shorter substrate. One interpretation of this difference is that the peptide products of the second substrate desorb from the protease more slowly due to more extensive hydrophobic interactions with the enzyme. Solowiej et al.^18^ found a similar Michaelis constant (*K*_a_ = 45 ± 4 μM) for (Dabcyl)KTSAVLQSGFRKME(Edans)-NH_2_ (pH 7.0) with SARS-CoV 3CL-PR, but with a larger turnover number: *k*_cat_ = 1.5 ± 0.2 s^–1^.

### pH- and pD-Rate Profiles and sKIEs

**Figure 1:**
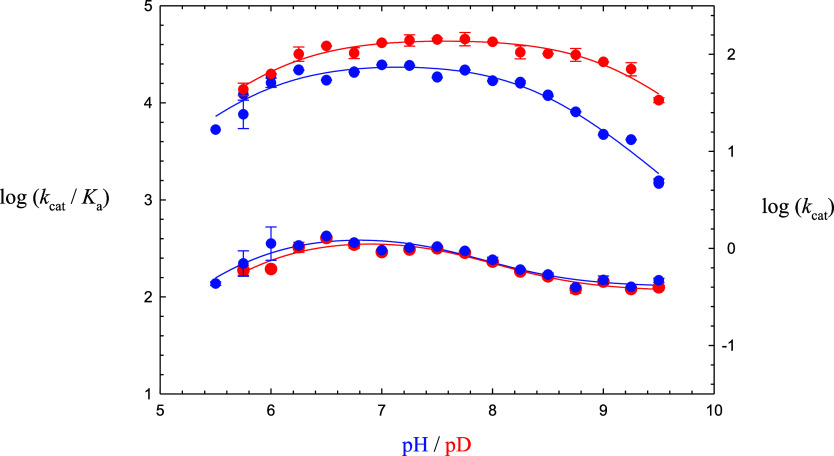
pH(D)-rate profiles for 3CL-PR-catalyzed cleavage of (Abz)SAVLQSGFRK(Dnp)-NH_2_ for which the kinetic parameters log(*k*_cat_/*K*_a_) vs pH (upper curve, blue) and pD (upper curve, red) and log(*k*_cat_) vs pH (lower curve, blue, and pD (lower curve, red) as shown. The curves drawn through the experimental data points are from fitting to eq 5 (log(*k*_cat_/*K*_a_) vs pH) or eq 6 log(*k*_cat_) vs pH. Averaged values of replicates and standard deviations are shown.

We ascertained the steady-state kinetic parameters of *k*_cat_/*K*_a_ and *k*_cat_ from 3CL-PR-catalyzed peptidolysis of (Abz)SAVLQ-SGFRK(Dnp)-NH_2_ at a range of pH values and pD values of 5.5–9.0. Shown in Figure 1 are plots of log(*k*_cat_/*K*_a_) vs pH (blue) and pD (red), which for both solvents conformed to apparent “bell-shaped” curves; that is, maximal values of *k*_cat_/*K*_a_ were found at pH(D) = 7.5–7.8, which decreased at lower values of pH(D) with apparent slopes = 1, and at higher values of pH(D) with apparent slopes = −1. Values of *k*_cat_/*K*_a_ in the plot of log(*k*_cat_/*K*_a_) vs pD are uniformly higher than that of log(*k*_cat_/*K*_a_) vs pH, with a slight rightward shift, as is commonly observed for pH(D) profiles.^35,41,42^ Fitting of both sets of data to eq 5 resulted in respective pH- and pD-independent values of *k*_cat_/*K*_a_ (*c*) of (2.6 ± 0.2) × 10^4^ M^–1^ s ^–1^ and (4.6 ± 0.2) × 10^4^ M^–1^ s ^–1^, which upon division of the former by the latter provided, with error propagation, the sKIE of ^D_2_O^(*k*_cat_/*K*_a_) = 0.56 ± 0.05 (Table 1). An inverse sKIE for *k*_cat_/*K*_a_ has been observed for other cysteine proteases including human cathepsin C,^25^ papain,^20^ and the trypanosomal cysteine protease, cruzain.^24^ In H_2_O and D_2_O, respectively, values of p*K*_1_ = 5.9 ± 0.1 and p*K*_2_ = 8.39 ± 0.07 and p*K*_1_ = 6.10 ± 0.07, and p*K*_2_ = 9.06 ± 0.06 were obtained from these fittings. Using (Dabcyl)KTSAVLQSGFRKME(Edans)-NH as substrate, pH-rate profiles for the 3CL-PR from SARS-CoV were reported to be p*K*_1_ = 6.25 ± 0.04 and p*K*_2_ = 8.3 ± 0.1,^18^ which are similar to what we have found here for SARS-CoV-2 3CL-PR (Abz)SAVLQSGFRK(Dnp)-NH_2_ as the substrate.

**Table 1:** pH-Rate Profiles and sKIEs from Steady-State Kinetics of 3CL Protease^a^

substrate	*k*_cat_/*K*_m_					sKIEs
	solvent	eqn fitted	p*K*_L_	p*K*_H_	*c* (M^–1^ s^–1^)	^D_2_O^*k*_cat_/*K*_m_
(*N*-Abz)Ser-Ala-Val-Leu-Gln*Ser-Gly-Phe-Arg-(N^e^-2,4-DNP)-Lys-NH_2_	H_2_O	5	5.9 ± 0.1	8.39 ± 0.07	(2.6 ± 0.2) × 10^4^	0.56 ± 0.05
	D_2_O	5	6.10 ± 0.07	9.06 ± 0.06	(4.6 ± 0.2) × 10^4^	

	*k*_cat_					sKIEs
	solvent	eqn fitted	p*K*_L_	p*K*_H_	*Y*_L_/*Y*_H_ (s^–1^)	^D_2_O^*k*_cat_
	H_2_O	6	5.6 ± 0.1	7.1 ± 0.2	1.4 ± 0.15/0.40 ± 0.03	1.0 ± 0.2/1.1 ± 0.1
	D_2_O	6	5.9 ± 0.1	7.7 ± 0.2	1.4 ± 0.2/0.37 ± 0.04	

dead-end inhibitor	solvent	eqn fitted	p*K*_L_	p*K*_H_	*K*_i_ (nM)	sKIEs
BC-666	H_2_O	5	6.5 ± 0.2	8.5 ± 0.1	620 ± 80	^D_2_O^*k*_3_ = 1.0 ± 0.3
						^D_2_O^*K*_i_ = 0.7 ± 0.2

affinity labels	solvent	eqn fitted	p*K*_L_	p*K*_H_	*k*_inact_/*K*_I_ (mM^–1^ min^–1^)
iodoacetamide	H_2_O	11	7.8 ± 0.1		*c* = 0.25 ± 0.04
diethyl pyrocarbonate	H_2_O	6	6.05 ± 0.07	7.6 ± 0.1	*Y*_L_ = 0.0069 ± 0.0005
					*Y*_H_ = 0.0015 ± 0.0001

a Data acquired at 25 °C in mixed buffers at pH(D) = 5.5–9.0. Errors displayed are the standard errors obtained from fitting to eqs 5, 6, and 11.

The fractionation factor of sulfhydryl groups is equal to 0.4–0.55, which means that the thiolate form of the sulfhydryl group of a catalytic cysteine residue, unlike other catalytic amino acids in which fractionation factors >1, is enriched in D_2_O by 2-fold or more.^37,43−45^ The different protonation/deuteriation states of Cys-SH, Cys-SD, HisH, HisD, HisH_2_^+^, and HisD_2_^+^ can be defined by the fractionation factors ϕ_SL_, ϕ_NL_, and ϕ_NL_^+^ as described in Schneck et al.^25^ and Belasco et al.^44^ as in eq 18
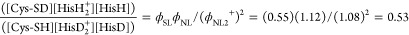
18

In D_2_O, the Cys-S^−^/Cys-SD ratio is 2-fold greater in the free enzyme form of 3CL-PR than the Cys-S^−^/Cys-SH ratio in H_2_O, which largely explains the inverse sKIE of D_2_O(*k*_cat_/*K*_a_) = 0.56 ± 0.05; there is a higher tautomeric fraction of Cys-S^−^ in D_2_O than in H_2_O, which results in the higher value of *k*_cat_/*K*_a_ in D_2_O. That is, the concentration of the more active imidazolium-thiolate form of the free enzyme (E^+/–^) is enhanced approximately 2-fold in D_2_O.

For the plots of log *k*_cat_ vs pH and pD, a decrease in the value of *k*_cat_ was present in either solvent at values of pL < 6.5, while at values of pL greater than the highest value of log *k*_cat_, a “wave-shaped” profile is observed (Figure 1). The lower values of *k*_cat_ at pH(D) > 7.0 (a 3.5–3.8-fold reduction) indicated that a change in rate-limiting step occurs when an enzymatic group of p*K*_2_ = 7.1 is deprotonated. Fitting these data to eq 6 resulted in pH(D)-independent values of *k*_cat_ at high and low pH(D), respectively, of *Y*_L_ = 1.4 ± 0.15 and *Y*_H_ = 0.40 ± 0.03 in H_2_O, and of *Y*_L_ = 1.4 ± 0.2 and *Y*_H_ = 0.37 ± 0.04 in D_2_O (Table 1).

Accordingly, there is a negligible sKIE on either value of *k*_cat_, suggesting that the rate-limiting step for 3CL-PR does not involve a proton-transfer step. From the fittings in H_2_O and D_2_O, respectively, values of p*K*_1_ = 5.6 ± 0.1 (H_2_O) and 5.9 ± 0.1 (D_2_O), and p*K*_2_ = 7.1 ± 0.2 (H_2_O) and 7.7 ± 0.2 (D_2_O) were obtained. A tentative assignment of the enzymatic group represented by p*K*_1_ could be the neutral imidazole group of His_41_, which must remain neutral in order to extract a proton from the lytic water molecule enabling hydrolysis of the thio-ester form of 3CL-PR. This value of p*K*_1_ = 5.6 is nearly equal to that of p*K*_1_ = 5.9 from the profiles of log(*k*_cat_/*K*_a_) vs pH(D), which we also ascribe to a neutral histidine residue. With regard to the diminution in *k*_cat_ observed at higher values of pH, deprotonation of an enzymatic group of p*K* = 7.1 lowers the value of the rate-limiting step. Accordingly, this enzymatic group does not participate in chemical catalysis, but upon its deprotonation, either a conformational change of the enzyme or increased affinity of the peptidolytic products, possibly due to enhanced hydrogen-bonding between enzyme and product, retards the nonchemical rate-limiting step. A combination of bell-shaped and wave-shaped pH-rate profiles for log(*k*_cat_/*K*_a_) and log(*k*_cat_), respectively, was also observed for HIV-1 protease^46^ and *Leuconostoc mesenteroides*(47) glucose 6-phosphate dehydrogenase, and attributed in both cases to a change in the rate of a postchemical step due to deprotonation of an enzymatic group. His-163 is a candidate to be this residue as it could exhibit a p*K* = 7.1, and its ε-nitrogen is within hydrogen-bonding distance to the side chain carbonyl oxygen of the glutamine substrate, while its δ-nitrogen is within hydrogen-bonding distance to Tyr-61.^46^ If Tyr-61 is protonated, deprotonation of this histidine above pH = 7.1 would strengthen the hydrogen-bonding of the δ-nitrogen with the phenol of Tyr-61.

### pH-Dependence of Competitive Inhibitor BC-666

**Figure 2:**
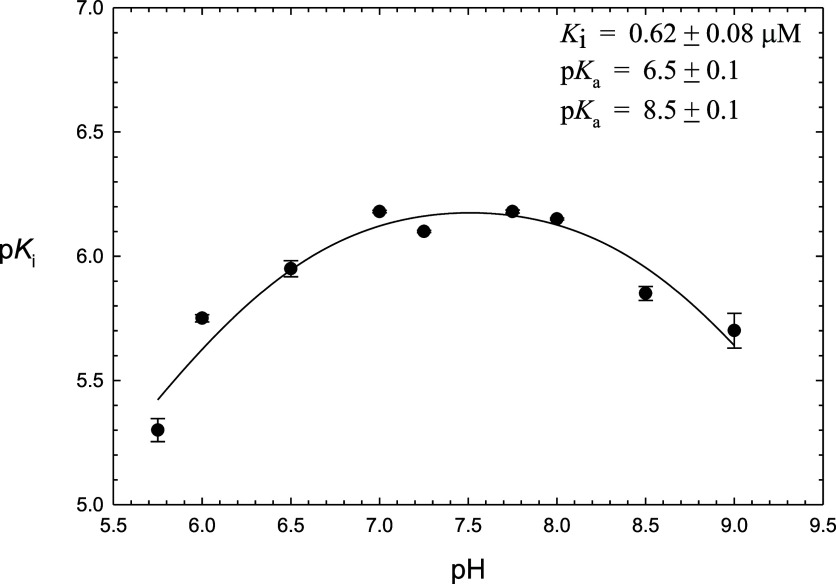
Profile of p*K*_i_ vs pH for the competitive inhibitor BC-666. The bell-shaped curve was fitted to eq 5, from which values of p*K*_i_, p*K*_1_, and p*K*_2_ as shown in the figure and in Table 1 were obtained. Averaged values of replicates and standard errors obtained from data fitting are shown.

The measurement of acid- and base-dissociation constants using the pH-rate profile of *k*_cat_/*K*_a_ often provides inaccurate values of p*K* when a substrate is sticky, that is, upon binding to enzyme its rate of desorption is equal to or less than the rate of its conversion to product.^38−40^ For such a substrate, even though the kinetic parameter *k*_cat_/*K*_a_ reports on the initial rate when [substrate] = >0, the enzymatic groups in the active site are not entirely accessible to protons and hydroxide ions in the reaction mixture, and p*K*_a_ and p*K*_b_ values are lowered and raised, respectively. A commonly used technique to circumvent this is to employ a competitive inhibitor, and determine its pH-dependence of inhibition from a plot of p*K*_i_ vs pH. Because a competitive inhibitor, which is generally in rapid equilibrium with the enzyme form to which it binds, provides a measure of the decrease of the value of *k*_cat_/*K*_a_, the pH-dependence of the inhibition constant can provide accurate values of acid- and base-dissociation constants for catalytic residues in the active site. We evaluated the pH-dependence of the inhibition constant of BC-666, a competitive inhibitor of 3CL-PR (Figure S16; *K*_i_ = 0.6 ± 0.1 μM (from initial rates). The plot of p*K*_i_ vs pH (5.5–9.0) for BC-666 conformed to a bell-shaped curve in which maximal inhibition (*K*_i_ = 0.62 ± 0.08 μM) was observed at pH ∼ 7.5 (Figure 2). Bell-shaped curves for competitive inhibitors are uncommon, as an inhibitor can often bind with different potency to a different protonation state of the enzyme.^38,40^ The similarities of the bell-shaped curves of log(*k*_cat_/*K*_a_) vs pH and p*K*_i_ vs pH could arise from the fact that the aldehyde BC-666 forms an apparent thiohemiacetal with Cys_145_ of 3CL-PR, which mechanistically resembles the acylation half-reaction as exemplified by the parameter *k*_cat_/*K*_a_. The acid- and base-dissociation constants obtained from fitting of this plot to eq 5 were p*K*_1_ = 6.5 ± 0.1 and p*K*_2_ = 8.5 ± 0.1, which correspond to the tentative p*K* values for histidine and cysteine, respectively, and likely comprise accurate values.

### Solvent Isotope Effect on Time-dependent Inhibition of 3CL-PR by BC-666

**Figure 3:**
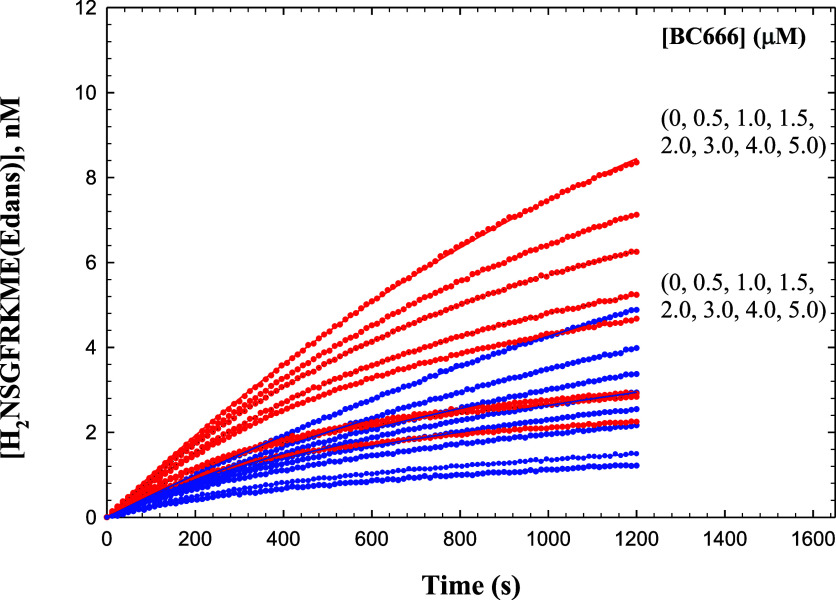
Time courses of inhibition of 3CL-PR by BC-666 in H_2_O (blue) and D_2_O (red). Each data point represents the average of two determinations (error bars included). The curves drawn through the experimental data were from fitting of each line using eq 8. Data for all concentrations of BC-666 for each solvent were fitting globally using eq 8 for which *k*_obs_ in eq 8 is substituted with eq 9.

We next evaluated the time-dependent inhibition of 3CL-PR by BC-666 in H_2_O and D_2_O at pL = 7.5. For the study in H_2_O, we included 9% (v/v) glycerol to provide a solution viscosity equivalent to that of 100% D_2_O (η_rel_ = 1.24).^34^ The assay was carried out at 30 μM substrate with inhibitor concentrations of 0–5 μM (Figure 3). In the absence of inhibitor, initial rates from time courses (*v*_i_) were greater in D_2_O than in H_2_O, as seen in the pH-rate profiles above (^D_2_O^*v*_i_ = 0.60 ± 0.02). Fitting of both sets of data to eq 8 in which the parameter *k*_obs_ is substituted with eq 9, provided negligible values of *k*_4_ (the rate constant for the conversion of the EI* complex to EI; Scheme 1), and values of *k*_3_ (the rate constant for the formation of the EI* complex from EI) and *K*_i_ were: *k*_3_ = 0.011 ± 0.002 s^–1^ (H_2_O) and *k*_3_ = 0.0104 ± 0.003 s^–1^ (D_2_O) and *K*_i_ = 8 ± 2 nM (H_2_O) and *K*_i_ = 14 ± 4 nM (D_2_O), leading to calculated solvent isotope effects of ^D_2_O^*k*_3_ = 1.0 ± 0.3 and ^D_2_O^*K*_i_ = 0.7 ± 0.2 after correction for the changes to the values of the Michaelis constants in the two solvents (Figure 3). These results demonstrated that the formation of the EI complex from its E and I components is enhanced by 30% in D_2_O, suggesting that the aldehyde inhibitor more favorably binds to the Cys-S^−^ enzyme form than the Cys-SH form, the former of which we have shown to be enriched in D_2_O. There is no apparent sKIE on the conversion of the EI complex to EI* (Scheme 1) (^D_2_O^*k*_3_ = 1.0 ± 0.3). The putative proton transfer in the *k*_3_ step of the mechanism of inhibition by BC-666 (Scheme 1) is apparently not expressed in this rate constant, possibly owing to the tight-binding of the EI complex.

### Native Mass Spectrometric Evaluation of the Interaction of the Peptide Aldehyde BC-666 with Wild-Type and the C145A Mutant of 3CL-PR

**Figure 4:**
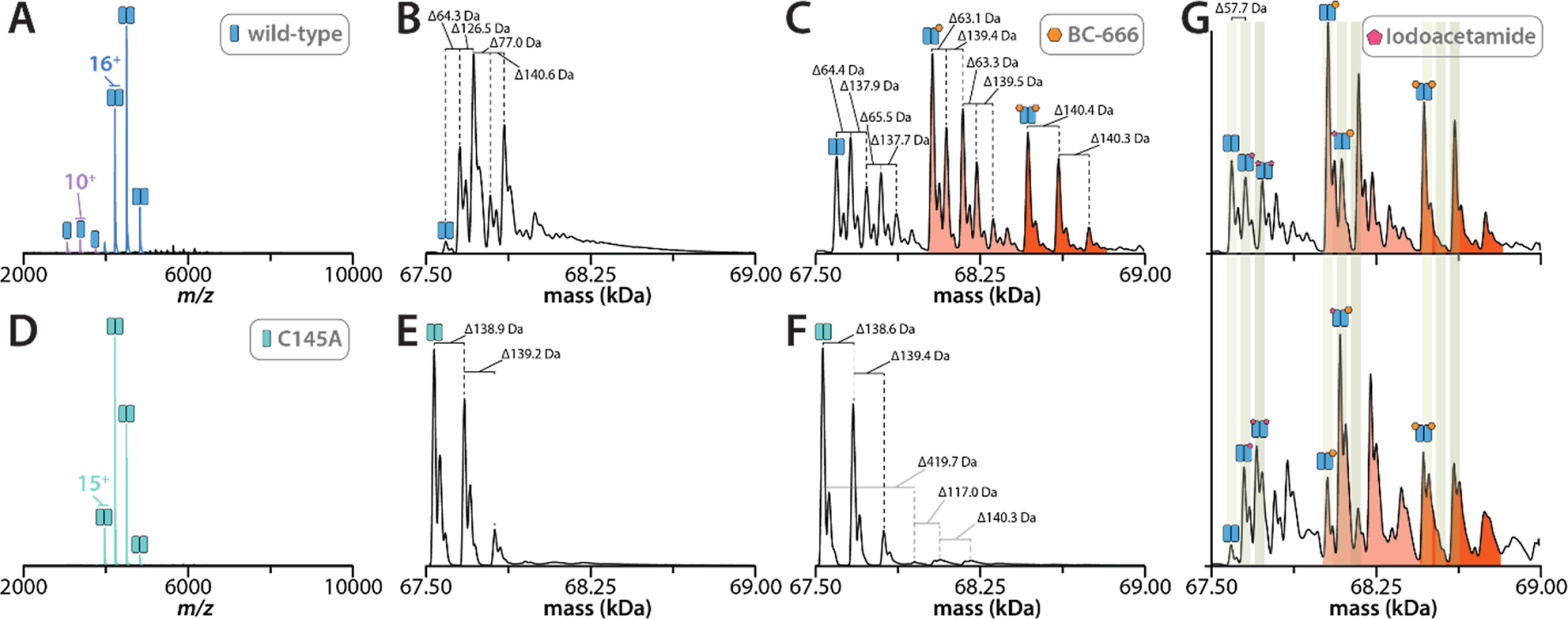
Native MS analysis of wild-type and C145A 3CL-PR. (A) Native mass spectrum of wild-type 3CL PR buffer exchanged into 200 mM ammonium acetate (pH 7.0), and deconvolution of the spectrum as shown in (B) (homodimer depicted as two blue rectangles). (C) Deconvoluted mass spectrum of the wild-type 3CL PR incubated with 5-fold molar excess of BC-666 (orange hexagon). Excess inhibitor not bound to the protein was removed during the buffer exchange. Peaks shaded light and dark orange depict species of the 67.6 kDa homodimer containing one and two molecules of bound BC-666, respectively, resulting in a molecular weight increase of +440 Da for each BC-666 bound. (D) Native mass spectrum of the C145A mutant of 3CL-PR following buffer exchange into 200 mM ammonium acetate (pH 7.0) and deconvolution of the spectrum shown in (E) (homodimer depicted as two teal rectangles). (F) Deconvoluted mass spectrum of C145A 3CL-PR incubated with a 5-fold molar excess of BC-666. The unbound excess inhibitor was removed during the buffer exchange. (G, top) Deconvoluted mass spectrum of wild-type 3CL-PR treated with 40-fold molar excess of iodoacetamide and a 5-fold molar excess of BC-666. The unbound excess ligands were removed during the buffer exchange. (G, bottom) Sample in (C) with a 40-fold molar excess of iodoacetamide added post buffer exchange.

As we were unable to obtain a crystal structure of BC-666 bound as the expected thiohemiacetal adduct, we employed native MS to investigate the binding of this peptide aldehyde inhibitor with both wild-type 3CL-PR and its C145A mutant (Figure 4). Upon incubation of wild-type 3CL-PR with a 5-fold molar excess of BC-666, followed by removal of excess inhibitor by buffer exchange, we observed apo (67.6 kDa), monoligand (67.6 kDa + 440 Da) and diligand (67.6 kDa + 880 Da) species of the 3CL-PR homodimers, with the monoligand protease species being the predominant form (Figure 4A–C). In contrast, only the apo form (67.6 kDa) of the homodimer of the C145A mutant of 3CL-PR was observed in a sample containing identical amounts of the C145A mutant 3CL-PR and a 5-fold molar excess of BC-666 (Figure 4D–F). One may conclude from these results that one molecule of BC-666 binds to one or both monomers of 3CL-PR that contains the active-site Cys_145_ with high affinity, and this would be the case when the thiohemiacetal adduct is formed. Next, we treated wild-type 3CL-PR with a 40-fold molar excess of iodoacetamide and a 5-fold molar excess of BC-666, followed by removal of unbound ligands by buffer exchange (Figure 4G, top). For a second sample containing wild-type 3CL-PR with a 5-fold molar excess of BC-666, a 40-fold molar excess of iodoacetamide (IAM) was added after the removal of BC-666 following buffer exchange (Figure 4G, bottom). One would expect the thiol-selective affinity agent, IAM, would compete with the peptide aldehyde for adduct formation with Cys_145_. In the first sample (Figure 4G, top), homodimers of wild-type 3CL-PR were observed that contained no, one, or two molecules of BC-666. For the apo 3CL-PR species (no inhibitor), nearly equimolar amounts of protein species of +57 or +114 Da were observed, arising from alkylation of Cys_145_ by IAM. These adducts were significantly reduced in the 3CL-PR-BC-666 and 3CL-PR-(BC-666)_2_ species, indicating that the bound BC-666 provides protection from alkylation by IAM. In the second sample in which BC-666 was removed prior to treatment with IAM (Figure 4G, bottom), for apo 3CL-PR, little of the unalkylated enzyme is present, and the +57 and +114 Da species in the unbound protease constituted the only observed species. For the 3CL-PR-BC-666 and 3CL-PR-(BC-666)_2_ proteases, little of the +57 Da species was observed, while the bis-alkylated +114 Da species were approximately equimolar to the unmodified 3CL-PR-BC-666 and 3CL-PR-(BC-666)_2_ enzyme forms. Accordingly, tight-binding of BC-666, including binding of the peptide aldehyde that survives buffer exchange, is able to reduce or prevent covalent inactivation of Cys_145_ by IAM. This provides compelling evidence that the 3CL-PR-BC-666 adduct comprises a thiohemiacetal adduct. We were unable to observe any adducts (+72 Da) of the wild-type 3CL-PR following treatment with DEPC, suggesting that any adducts formed were unstable in native MS analysis, as has been reported for *N*-carboxyethylated histidine residues in DEPC-treated protein.^49^

### pH-Dependence of Affinity-Labeling by Iodoacetamide and Diethylpyrocarbonate

**Figure 5:**
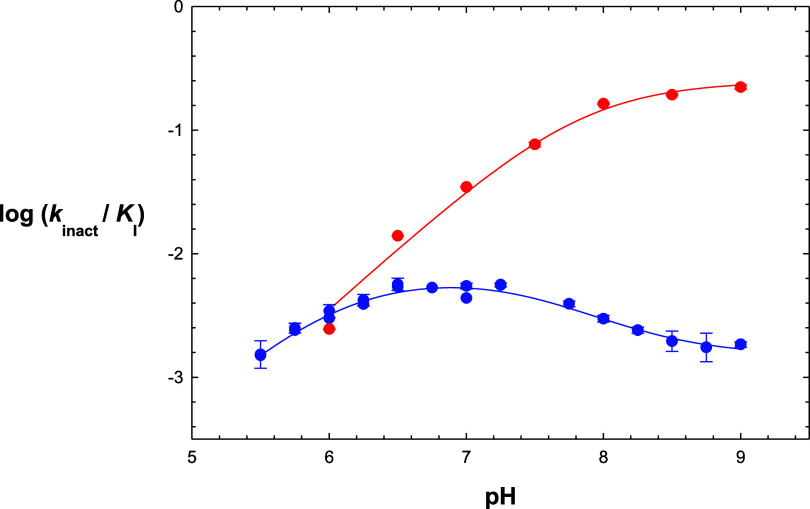
Plots of log(*k*_inact_/*K*_I_) vs pH for inactivation of 3CL-PR by iodoacetamide (red) and diethylpyrocarbonate (blue). The curves drawn through the experimental data points were from fitting to eq 11 (IAM) and eq 6 (DEPC) from replicate determinations (standard errors (bars) are shown).

In further studies to determine the true p*K* values of Cys_145_ and His_41_, we investigated the pH-dependence of covalent affinity labeling using cysteine- (iodoacetamide, IAM) and histidine- (diethypyrocarbonate, DEPC) specific agents. Iodoacetamide (0–1 mM) was preincubated with 2 μM 3CL-PR for 0–40 min at pH values of 6–9. Aliquots were removed and diluted 100-fold into reaction mixtures containing substrate at pH = 7.5, and residual enzyme activity was measured as *v*_i_/*v*_0_. Plotting of the residual enzyme activity log(*v*_i_/*v*_0_) vs time at different concentrations of IAM provided values of *k*_obs_ of inactivation by fitting to eq 10 (Figure S17). A plot of log(*k*_inact_/*K*_I_) vs pH (Figure 5) is characterized by a plateau at high pH, which, upon apparent protonation of an enzymatic residue of p*K* ∼ 8 resulted in diminution of inactivation with an apparent slope of 1. Fitting of this plot to eq 11 provided values of *c* = 0.25 ± 0.04 mM^–1^ s^–1^ and p*K*_1_ = 7.8 ± 0.1. As inactivation of 3CL-PR by IAM results from alkylation Cys_145_ as shown above, the p*K* value represents conversion of the thiolate form of Cys_145_ to the less reactive thiol form at pH < 7.8. These results confirmed that the p*K* value of 7.8–8.5 observed in all pH-rate data represents Cys_145_. A similar pH-rate profile for the inactivation of SARS-CoV 3CL-PR^18^ was reported, in which the investigators found an identical value of p*K*_1_ = 7.8 ± 0.1, indicating that the p*K* values of Cys_145_ of the 3CL-PRs from both SARS-CoV and SARS-CoV-2 are the same.

In kind, we evaluated the time-dependent inactivation of 3CL-PR with diethylpyrocarbonate (DEPC) at pH = 5.5–9.0 (example data found in Figure S18). Values of *k*_obs_ vs [DEPC] were obtained from a plot of log(*v*_i_/*v*_0_) vs time for each concentration of DEPC with fitting of data to eq 10. The resulting replots of *k*_obs_ vs [DEPC] were apparently linear (Figure S18), and plotting of the slopes (*k*_inact_/*K*_I_) vs pH conformed to a “wave-shaped” curve in which values of *k*_inact_/*K*_I_ decreased below pH ∼ 7 with an apparent slope of 1, and at higher pH shifted to a lower value above pH ∼ 7.5 (Figure 5). These data were fitted to eq 6, from which we obtained parameters of: p*K*_1_ = 6.05 ± 0.07, p*K*_2_ = 7.6 ± 0.1, *Y*_L_ = 0.0069 ± 0.0005 mM^–1^ s^–1^ and *Y*_H_ = 0.0015 ± 0.0001 mM^–1^ s^–1^. Protonation of an enzymatic group of p*K*_1_ = 6.05 resulted in ablation of inactivation by DEPC. This p*K* likely represents His_41_, which upon protonation is no longer amenable to *N*-acylation by DEPC. From the data summarized in Table 1, the p*K* value of His_41_ is therefore 6.0–6.5. For the value of p*K*_2_ = 7.6, which represents the reduction of *k*_inact_/*K*_I_ at higher pH, it is likely that this p*K* represents Cys_145_, so that the rate of inactivation of 3CL-PR by DEPC is highest when both Cys_145_ and His_41_ are neutral. We speculate that the observed decrease in *k*_inact_/*K*_I_ at pH > 7.6 in which Cys_145_ becomes increasingly deprotonated may lead to a second, competing mechanism of covalent inactivation, that is, formation of *S*-carboethoxy-Cys_145_. Such an adduct would be recognized as the acyl-enzyme intermediate and undergo rapid His_41_-catalyzed hydrolysis to produce catalytically competent free 3CL-PR, which would explain a wave-shaped profile rather than complete loss of enzyme activity.

We conclude from the steady-state kinetic studies: (1) Cys_145_ and His_41_ exist as neutral forms with respective values of p*K* = 8.2 ± 0.4 and 6.2 ± 0.3 in apo 3CL-PR (averaging of all p*K* values from all plots found in Table 1). (2) In D_2_O, the charged, thiolate-imidazolium catalytic tautomer predominates for the free enzyme, which accounts for the large, inverse sKIE of D_2_O(*k*_cat_/*K*_a_) = 0.56 ± 0.05. (3) The p*K* value of 5.6 observed in the plot of log *k*_cat_ vs pH(D) likely represent the neutral form of His_41_, which is needed to extract a proton from the lytic water molecule during the deacylation half-reaction. The absence of an sKIE on *k*_cat_ indicated that a reaction step not involving a proton transfer is rate-limiting to overall catalysis.

X-ray crystal structures of SARS-CoV-2 3CL-PR at 1.8 Å resolution also suggested that the neutral Cys_145_-SH-His_41_ catalytic dyad exists in the apo form of the protease,^50,51^ in accord with our kinetic data. However, these findings are at variance with structural data derived from neutron diffraction data of ligand-free^52^ and inhibitor-bound^53^ SARS-CoV-2 3CL-PR by Kneller and colleagues. In their studies, in the free enzyme, the active-site residues Cys_145_ and His_41_ conclusively appear as an imidazolium-thiolate pair, while in the inhibitor-bound enzyme, Cys_145_ forms a thiohemiketal adduct with the ketoamide group of the inhibitor, while His_41_ is unprotonated, presumably following proton transfer to the thiohemiketal oxygen.

How can these differences be reconciled? Neutron diffraction data are acquired in deuterium oxide, in which we have shown that the Cys_145_-S^–^-His_41_H^+^ tautomer of the free enzyme predominates, as indicated by the observed inverse sKIEs on both peptidolysis and binding of a peptide aldehyde. The enrichment of the fraction of free enzyme that contains the thiolate form of Cys_145_-S^–^ in D_2_O results in faster rates of *S*-acylation and the formation of the thiohemiacetal of BC-666, in accord with the neutron diffraction data. BC-666 contains the same C-terminus and similar peptide side chains as LL-478, which we showed by X-ray crystallography to form a thiohemiacetal adduct with the active-site Cys_145_ of 3CL-PR.^54^ Similar inverse sKIEs were also reported for SARS-CoV 3CL-PR^18^ in which the FRET-based substrate (Dabcyl)KTSAVLQSGFRKME(Edans)-NH was studied. Under-steady-state conditions, values of ^D_2_O^(*k*_cat_/*K*_a_) = 0.37 ± 0.06 and ^D_2_O^*k*_cat_ = 0.39 ± 0.05 were found for this substrate and SARS-CoV 3CL-PR, which differs from our data with a different substrate primarily in that we see no sKIE on the value of *k*_cat_. The equivalence of ^D_2_O^(*k*_cat_/*K*_a_) and ^D_2_O^(*k*_cat_) as observed by Solowiej et al.^18^ would be expected in a case in which the acylation half-reaction is equal to or slower than the rate of the deacylation, since sKIEs on *k*_cat_ are normal when the rates of deacylation are significantly slower than the rates of acylation.

### Pre-steady-state Analysis of the 3CL-PR-Catalyzed Peptidolysis of (Abz)SAVLQ*SGFRK(Dnp) and (Dabcyl)KTSAVLQ*SGFRKME(Edans)-NH_2_

We employed pre-steady-state kinetics to investigate whether the acylation and deacylation half-reactions of 3CL-PR-catalyzed peptidolysis of (Abz)SAVLQ*SGFRK(Dnp) could be observed and quantified by stopped-flow fluorimetric analysis of the Abz-SAVLQ-COOH product (designated as Q).

**Figure 6:**
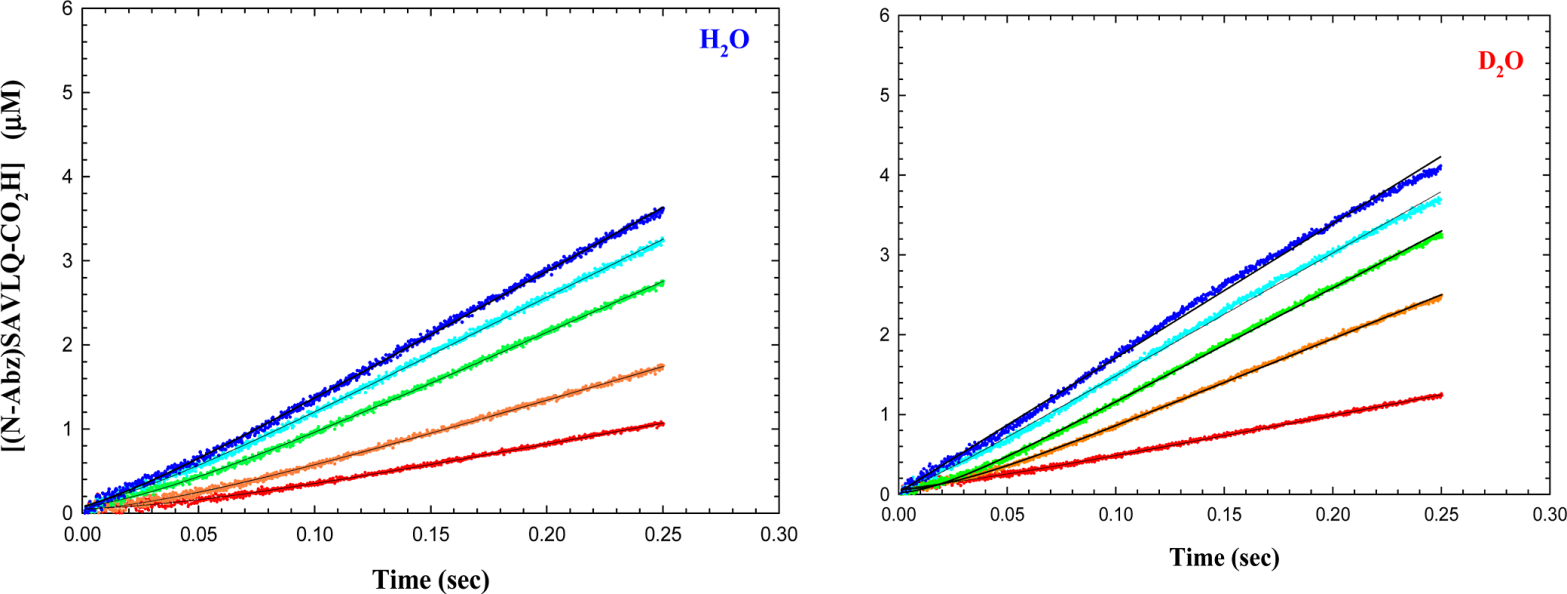
Presteady-state time courses of 3CL-PR-catalyzed hydrolysis of (Abz)SAVLQSGFRK(Dnp) (15 (red), 30 (orange), 60 (green), 90 (cyan), and 120 (blue) μM) in H_2_O and D_2_O at 0.002–0.250 s. Data in the time courses represent six or more replicates each. The black lines drawn through the experimental data at each substrate concentration represent fitting of data to eq 12. By fitting each time course to eq 12, we obtained individual values of *k*_ss_ and λ at each concentration of substrate as shown in Figure S19. Amplitudes in these plots were undeterminable. For the two replots of time-course data, apparent values of *k*_ss_ and λ increased with increasing substrate concentrations, and for both parameters, values were higher in D_2_O compared to H_2_O.

3CL-PR-catalyzed hydrolysis of (Abz)SAVLQ*SGFRK(Dnp) (15–120 μM) in both H_2_O and D_2_O were generated at pH(D) = 7.5 in a stopped-flow fluorimeter, by measurement of product (Abz)SAVLQ-CO_2_H at 2–200 ms. The other product, H_2_N-SGFRK(Dnp) (P) could not be evaluated by fluorescence. For each substrate concentration in either solvent, all time courses exhibited a transient rate (λ) characterized by an initial lag in formation of the amine product, followed by the establishment of a linear, steady-state rate of product formation (*k*_ss_) (Figure 6).

**Figure 7:**
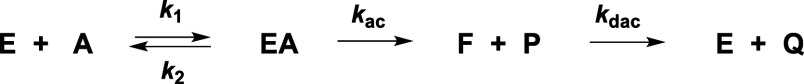
Minimal kinetic scheme for the double-displacement mechanism of 3CL-PR. Binding of substrate A to free enzyme is characterized by the dissociation constant, *K*_ia_ = *k*_2_/*k*_1_, the rate constant for acylation of the protease and release of the anime product P is given by *k*_ac_, and the rate of deacylation of the acyl-protease (F) to produce the carboxylic product (Q) is given by *k*_dac_.

Both sets of data were fitted globally to eq 16, from which we obtained the data found in Table 2. As with cathepsin C^25^ and cruzain,^24^ the transient rates likely correspond to the rates of acylation of 3CL-PR (*k*_ac_ in Figure 7) and were found to be *k*_acΗ_2_O_ = 42 ± 1 s^–1^ and *k*_acD_2_O_ = 78 ± 3 s^–1^.

**Table 2:** Pre-steady-state Kinetic Parameters of 3CL Protease-Catalyzed Hydrolysis of (Abz)SAVLQ*SGFR(Dnp)K-NH_2_ in H_2_O and D_2_O^a^

substrate	solvent	eqn fitted	*k*_ac_ (s^–1^)	*k*_dac_ (s^–1^)	*k*_cat_ (s^–1^)	*K*_a_ (mM)
(*N*-Abz)Ser-Ala-Val-Leu-Gln-Ser-Gly-Phe-Arg-(N^ε^-2,4-DNP)-Lys-NH_2_	H_2_O	16	42 ± 1	5.91 ± 0.03	5.2 ± 0.2	82.1 ± 0.5
	D_2_O	16	78 ± 3	5.89 ± 0.04	5.0 ± 0.4	53.2 ± 0.3

solvent kinetic isotope effects	^D_2_O^*k*_ac_	^D_2_O^*k*_dac_	^D_2_O^*k*_cat_	^D_2_O^*K*_a_
	0.54 ± 0.02	1.00 ± 0.002	1.04 ± 0.09	1.54 ± 0.01

a Data obtained at room temperature and pH(D) 7.5.

This resulted in an inverse sKIE on the rate of acylation of ^D_2_O^*k*_ac_ = 0.54 ± 0.02, nearly identical to that determined for the steady-state parameter ^D_2_O^(*k*_cat_/*K*_a_) = 0.56 ± 0.05. In H_2_O, the rate of deacylation (*k*_dac_ = 5.91 s^–1^) is slightly greater than the steady-state rate value of (*k*_cat_ = 5.2 s^–1^). This is self-consistent with the calculation: *k*_cat_ = *k*_ac_*k*_dac_/(*k*_ac_ + *k*_dac_) = (42)(5.91)/(42 + 5.91) = 5.2 s^–1^. Likewise, in D_2_O, the rate of deacylation (*k*_dac_ = 5.89 s^–1^) is slightly greater than the steady-state rate value of (*k*_cat_ = 5.2 s^–1^). In agreement with the steady-state sKIE data, there is no appreciable solvent kinetic isotope effect on *k*_dac_ or *k*_cat_. Again in D_2_O, *k*_cat_ = *k*_ac_*k*_dac_/(*k*_ac_ + *k*_dac_) = (78)(5.89)/(78 + 5.89) = 5.0 s^–1^. Respective values of *K*_aH_2_O_ = 82.6 ± 0.1 μM, *K*_aD_2_O_ = 62.7 ± 0.5 μM, and ^D_2_O^*K*_a_ = 1.54 ± 0.01 were obtained (Table 2).

**Figure 8:**
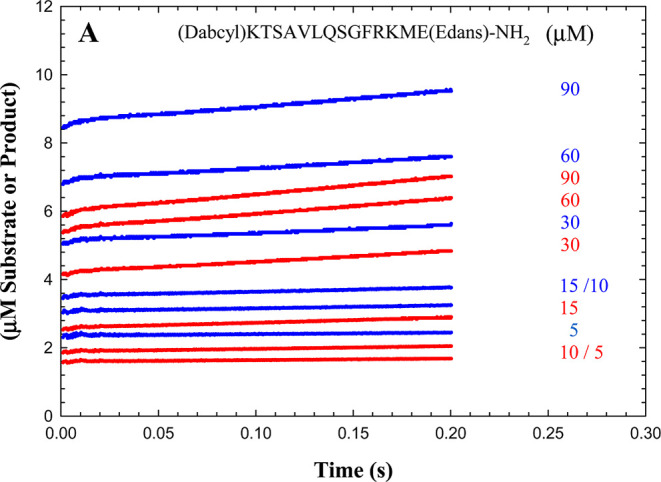
(A) Pre-steady-state time courses of 3CL-PR-catalyzed hydrolysis of (Dabcyl)KTSAVLQSGFRKME(Edans)-NH_2_ (15–90 μM) in H_2_O (blue) and D_2_O (red) at 0.002–0.2 s. Kinetic parameters were obtained by fitting data globally to eq 17.

In order to investigate whether or not a presteady burst would be observed with a substrate for which the fluorescent product is the amine product, we acquired pre-steady-state kinetic data for the 3CL-PR-catalyzed hydrolysis of (Dabcyl)KTSAVLQ*SGFRKME(Edans)-NH_2_ (5–90 μM) in both H_2_O and D_2_O at pH(D) = 7.5 in a stopped-flow fluorimeter, by measurement of fluorescence at 520 nm at 2–200 ms (Figure 8). In either solvent, time courses exhibited very rapid transient rates that had apparently been completed at <2 ms, and burst amplitudes were observed as *t* → 0, and were dependent on the concentration of substrate. These burst amplitudes were clearly higher in H_2_O than in D_2_O. Data for substrate concentrations of 15–90 μM and at 10 μM 3CL-PR (those in excess of the enzyme concentration of 10 μM) were fitted globally to eq 17, with the following results (steady-state parameters; *t* = 2–200 ms): (H_2_O) *k*_cat_ = 0.469 ± 0.007 s^–1^, *K*_a_ = 43 ± 2 μM, *k*_cat_/*K*_a_ = (10.9 ± 0.5) × 10^4^ μM^–1^ s^–1^, and (D_2_O): *k*_cat_ = 0.91 ± 0.02 s^–1^, *K*_a_ = 46 ± 3 μM, *k*_cat_/*K*_a_ = (20 ± 1) × 10^4^ μM^–1^ s^–1^; and burst amplitudes:  = 11 ± 1 μM, *K*_ia_ = 28 ± 7 μM, (H_2_O); and  = 7.9 ± 0.5 μM, *K*_ia_ = 28 ± 4 μM, (D_2_O). As mentioned, in both solvents, the transient values were too rapid for them to be evaluated. Steady-state initial rates were evident, which apparently increased at increasing substrate concentrations.

The finding that burst amplitudes were established in less than 2 ms, were dependent on the input concentration of the substrate, and were less than or equal to the concentration of 3CL-PR (10 μM) indicated that the observed fluorescence resulted from uncleaved substrate in which the quenching group is spatially displaced from the incipient fluorescent group. We observed similar results when the final substrate and 3CL-PR concentrations were, respectively, 12.5 and 1 or 0.1 μM. We propose that this results from the binding of the peptide substrate (Dabcyl)KTSAVLQ*SGFRKME(Edans)-NH_2_ to 3CL-PR, and the “linearization” of the tetradecapeptide in the active site sufficiently displaced the quenching Dabcyl group from the Edans fluor upon binding. A previous study with this FRET-based substrate and SARS-CoV-2 3CL-PR (both the wild type and the WT and Cys_145_Ala mutant) both showed rapid transients with measured on-rates of 2.6–2.8 × 10^6^ s^–1^.^55^ Accordingly, the observed “dead-time” burst amplitude provided quantification of the fraction of substrate-bound enzyme, as discussed below. These authors also concluded that the “stretching out” of the FRET-based peptide upon binding to form the active (or inactive in the case of the mutant) was a potential cause of these burst amplitudes.^55^ Accordingly, the fluorescence observed in Figure 8 reflects substrate binding and not acylation. The dissociation constant (*K*_ia_ = 28 μM) for (Dabcyl)KTSAVLQ*SGFRKME(Edans)-NH_2_ was identical in both H_2_O and D_2_O, but the concentration of [E-substrate; EA] (extrapolated to infinite substrate concentration) was EA = 11 ± 1 μM and 7.9 μM, in H_2_O and D_2_O, respectively. That [EA] is 14% larger in H_2_O than in D_2_O is consistent with the expected 14% greater solution viscosity in D_2_O (η_rel_ = 1.24), which retard the binding kinetics of the substrate to free enzyme.

In order to provide additional evidence for this, we repeated some of the studies of Zakharova et al.,^55^ for which we prepared the C145A mutant of SARS-CoV-2 3CL-PR, and conducted additional pre-steady-state studies using the (Dabcyl)KTSAVLQ*SGFRKME(Edans)-NH_2_ substrate, and compared the pre-steady-state time courses alongside that of the wild-type enzyme. At three concentrations of this substrate, both wild-type and C145A 3CL-PR at 10 μM concentrations exhibited pre-steady-state burst amplitudes of 0.25, 4.2, and 9.0 μM at 5, 30, and 60 μM substrate, respectively (Figure S20), following very rapid transient rates as seen in Figure 8. As above, the burst amplitudes for both proteases increased commensurately with the fixed substrate concentration, indicating that 90% of either enzyme has bound substrate at 90 μM concentrations. However, only in the time courses of the wild-type 3CL-PR were postburst, steady-state rates of peptidolysis observed, as the time courses with the C145A mutant protease had near-zero slopes as expected for the noncatalytic mutant protease. These results support our proposal that the pre-steady-state burst amplitudes observed with the (Dabcyl)KTSAVLQ*SGFRKME(Edans)-NH_2_ substrate are due to peptide binding, and not scission of the substrate upon acylation of the protease.

The sKIEs calculated from the steady-state initial rates from the stopped-flow data were: ^D_2_O^(*k*_cat_/*K*_a_) = 0.6 ± 0.2 and ^D_2_O^*k*_cat_ = 0.50 ± 0.01. The observation of identical inverse sKIEs for ^D_2_O^(*k*_cat_/*K*_a_) and ^D_2_O^*k*_cat_ for (Dabcyl)KTSAVLQ*SGFRKME(Edans)-NH_2_ differs from that of (Abz)-SAVLQ*SGFRK(Dnp) for which ^D_2_O^(*k*_cat_/*K*_a_) = 0.56 ± 0.05 and ^D_2_O^(*k*_cat_) = 1.0 ± 0.1. The common values of ^D_2_O^(*k*_cat_/*K*_a_) = 0.6 for both substrates again indicated that the more catalytic tautomer of 3CL-PR (Cys-S^–^-HisH^+^) is enriched in D_2_O, resulting in an enhanced value of the rate of the acylation half-reaction, as reported by the *k*_cat_/*K*_a_ parameter.

The finding that ^D_2_O^(*k*_cat_) = ^D_2_O^(*k*_cat_/*K*_a_) = 0.6 for (Dabcyl)KTSAVLQ*SGFRKME(Edans)-NH_2_ would be observed in the case in which the acylation half-reaction is slower than the subsequent deacylation half-reaction. This would account for the lack of observation of a second exponential phase in the pre-steady-state data, that is a second burst with an observable transient rate, as was observed for cruzain^24^ and cathepsin C.^25^

Unlike our data, Solowiej et al.^18^ observed a pre-steady-state burst and a transient rate with a single concentration of the substrate (Dabcyl)KTSAVLQ*SGFRKME(Edans)-NH_2_ for which the transient rates were 50 s^–1^ and 24 s^–1^ in H_2_O and D_2_O, respectively, and which most likely represent the rates of acylation. The normal sKIE on the rate of acylation (^D_2_O^*k*_ac_ = 1.8 s^–1^) differs from our finding that the transient lag rates for the other substrate (Abz)SAVLQ*SGFRK(Dnp)-NH_2_ were *k*_acΗ_2_Ο_ = 42 ± 1 s^–1^ and *k*_acD_2_Ο_ = 78 ± 3 s^–1^, but apart from that, the earlier data represent a single substrate concentration while ours extrapolate to substrate saturation.

### Catalytic Mechanism of SARS-CoV-2 3CL-PR

Our kinetic data of 3CL-PR as reported here for the substrate (Abz)SAVLQSGFRK(Dnp)-NH_2_ are consistent with the following conclusions:

(1) The p*K* values of the active-site Cys_145_ and His_41_ groups were, respectively, p*K* = 8.2 ± 0.4 and p*K* = 6.2 ± 0.3. The predominant tautomer at neutral pH of 3CL-PR in H_2_O is the neutral form of the catalytic dyad (Cys-SH--His).
(2) In D_2_O, the charged, thiolate-imidazolium catalytic dyad predominates for the free enzyme, which accounts for the large, inverse sKIE of ^D_2_O^(*k*_cat_/*K*_a_) = 0.56 ± 0.05. This is consistent with the observation of the thiolate-imidazolium form of the free enzyme in neutron diffraction studies.^52,53^ The absence of a sKIE on ^D_2_O^*k*_cat_ = 1.00 ± 0.002 indicates that the chemical steps of the deacylation half reaction are not the rate-limiting step(s) of turnover of this substrate.
(3) The pre-steady-state time courses of (Abz)SAVLQ*SGFRK(Dnp)-NH_2_ at multiple concentrations were characterized as pre-steady-state lags followed by steady-state rates, which were considerably faster in D_2_O than in H_2_O (^D_2_O^*k*_ac_ = 0.54 ± 0.02). We believe that the transient rate encompasses substrate binding and the steps of acylation (*k*_3_ and *k*_5_), which are faster in D_2_O because the more active Cys-S^–^-HisH^+^ tautomer is enriched in D_2_O.

**Figure 9:**
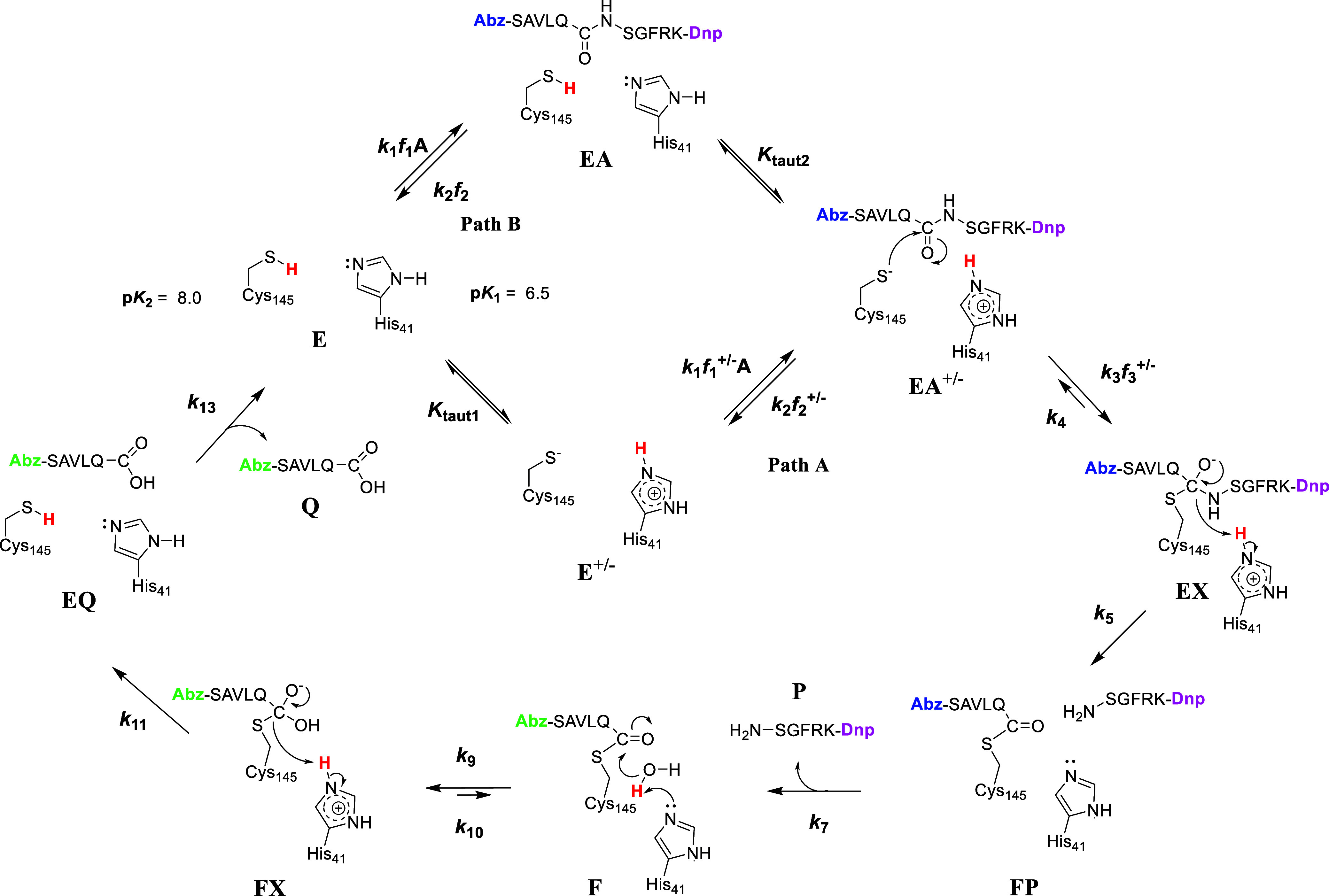
Proposed catalytic mechanism for SARS-CoV-2 3CL-PR for the FRET-based substrate (Abz)SAVLQSGFRK(Dnp)-NH_2_ based on (pre)-steady-state kinetic studies. Protons expected to contribute to primary sKx IEs are in bolded red, and the predominant free enzyme species in H_2_O and D_2_O are within respective blue and red rectangles. The recoloring of the 2-aminobenzoyl group from blue to green color and the dinitrophenyl group from magenta to black indicates the formation of fluorescent species when separated from the quenching group Dnp. The kinetic parameters for *k*_cat_/*K*_a_ include the acylation kinetic steps *k*_1_–*k*_7_, while for *k*_cat_, the deacylation kinetic steps *k*_9_–*k*_13_. In Path A, defined by *k*_1_*f*_1_^+/–^, substrate A only binds to the imidazolium-thiolate form of 3CL-PR (E^+/–^), while in the Path B pathway defined by *k*_1_*f*_1_, A binds to the imidazole-thiol tautomer (E). The pathways described from QM/MM modeling studies^29,30^ are included in Figure S21, and Figure S22 is a black-and-white version of the figure, which also includes a scheme for the binding of peptide aldehyde inhibitor BC-666.

In Figure 9 are shown potential chemical mechanisms of SARS-CoV-2 3CL-PR based on our data herein. The figure depicts that substrate (Abz)SAVLQSGFRK(Dnp)-NH_2_ (A) could bind to either the neutral free enzyme form E, (*f*_1_), to the fraction of free enzyme that is in the imidazolium-thiolate form, E^+/–^ (*f*_1_^+/–^), or both tautomers. In the case where substrate binds to free enzyme E, proton transfer from Cys_145_-SH to His_41_ in the EA complex leads to the thiolate-imidazolium form EA^+/–^. The thiolate of Cys_145_ then adds to the substrate carbonyl carbon to produce tetrahedral intermediate EX. Proton transfer from His_41_H^+^ to the amide nitrogen precedes C–N bond breakage to afford the *S*-acylated protease F and the fluorescent amine product P. It is likely that the quenching group Dpn on the H_2_N-SGFRK(Dnp)-NH_2_ product in the FP complex is sufficiently proximal to the fluorescent group Abz in the acyl-enzyme group E-S-C(O)-QLVAS(Abz) to continue to quench its fluorescence despite C–N bond cleavage. In this case, desorption of the amine product P completes the acylation half-reaction of the mechanism and gives rise to the observed fluorescence at the end of the pre-steady-state lag phase. Neutral His_41_ then deprotonates bound water sponsoring hydroxide addition to the carbonyl carbon of the acyl-enzyme F, leading to the second tetrahedral intermediate, FX. This intermediate then collapses to provide the carboxylic acid product (Q), which upon its release restores 3CL-PR to its neutral form E. If product release is slower than the chemistry of the deacylation half-reaction, one would not observe an sKIE on *k*_cat_, as was observed.

Other chemical mechanisms have been proposed from quantum mechanical/molecular modeling studies. Ramos-Guzmán et al. conducted molecular modeling studies of SARS-CoV-2 3CL-PR in which the modeled peptide substrate was Ac-Ser-Ala-Val-Leu-Gln-Ser-Val-Gly-Phe-NMe. They concluded that the enzyme underwent acylation via an asynchronous mechanism in which formation of the thiolate-imidazolium catalytic dyad follows substrate binding (EA → E^+/–^), and His_41_-mediated protonation of the amide nitrogen of the P_1′_ Ser group precedes attack of the thiolate of Cys_145_ in a concerted step in which a discrete intermediate such as that in EX does not form (Figure S21).^29^ These authors also conclude that the N-terminal amino group of the enzyme bound P product H_2_N-Ser-Val-Gly-Phe-NMe, and not His_41_, is the general base that deprotonates the water molecule prior to its attack on the acyl-enzyme, and this is discussed below.

In the molecular modeling studies of Świderek and Moliner using the substrate Ac-Val-Lys-Leu-Gln-ACC, the authors proposed a stepwise acylation mechanism in which (a) the substrate also binds to the neutral catalytic dyad form E of the enzyme and (b) proton transfer from Cys_145_-SH to His_41_ is concomitant with attack of the incipient thiolate on the carbonyl oxygen of the substrate (EA to EX in Figure S21). The authors concluded that this catalytic step is rate-limiting in the acylation half-reaction. Protonation of the amide nitrogen of the substrate by His_41_ occurs after formation of the tetrahedral intermediate, as we also depict for EX in Figure 9.^30^ As noted in Ramos-Guzmán et al., the different outcomes of these molecular modeling studies may depend on the choice of substrate, in which the former study involves an octapeptide substrate encompassing the P_4_–P_4^′^_ residues, while the latter employs a peptide encompassing authentic P_4_–P_1_ residues but with an 4-amino-7-methylcarbamoyl-coumarin (ACC) moiety replacing the serine at the P_1^′^_ position.

Outcomes of our kinetic studies differ significantly from these molecular modeling studies. (1) While our studies indicated that the neutral free enzyme form E is the predominant species in H_2_O, the observed inverse sKIEs can only be due to the binding of substrate A to the E^+/–^ form of the free enzyme because this is the major free enzyme form in D_2_O (Figure 9, Path A). (2) Formation of the putative EX complex in our mechanism via the *k*_3_ step does not involve proton transfer unlike the EA → EX step proposed by Świderek and Moliner, which they ascribe as rate-limiting for the acylation half-reaction (Figure S21). In our case, this simpler chemical step (no proton transfer) is not likely to be rate-limiting, as indicated by data in Table 3. (3) There is disagreement in the two modeling studies as to whether or not the tetrahedral intermediate species in the EX complex is a discrete intermediate. Ramos-Guzmán et al. concluded that there is no EX intermediate, and their proposed mechanism would be represented by steps EA → E^+/–^A → FP in (Figure S21, Path C), while the pathway proposed by Świderek and Moliner includes this intermediate and is depicted as EA → EX → FP (Figure S21, Path B). Ramos-Guzmán et al. do propose that a tetrahedral intermediate does form in the deacylation half-reaction (FX). As the existence of the EX species is unresolved by the two sets of molecular modeling studies, we have no reason to exclude it from our proposed mechanism. (4) Ramos-Guzmán et al. concluded that the amine product peptide H_2_N-SGFRK-Dnp not only remains bound to 3CL-PR following its C–N bond cleavage, but that its N-terminal amino group, and not His_41_, is the general base that deprotonates the lytic water in the first step of the deacylation half-reaction. This would require that the bound H_2_N-SGFRK-Dnp remains proximal to the Abz group in the acyl-enzyme species (E-S-C(O)-QLVAS-Abz) (Figure S21). Under these circumstances, our observed pre-steady-state lag would arise only after the separation of P and Q upon the desorption of one product from the EPQ complex. Importantly, the general base role of bound H_2_N-SGFRK described by Ramos-Guzmán et al.^29^ is at variance with the crystal structure data of an acylated form of 3CL-PR in which the Nε2 nitrogen of His_41_ is within 2.9 Å of the putative water lytic molecule, which itself is within 2.7 Å from the carbonyl carbon of the SGVTFQ-C(O)-S-Cys thioester.^50^ Given the disparities among these mechanisms, we retain the EX complex in our mechanism although it may be fleeting, and we propose that the H_2_N-SGFRK-Dnp product desorbs prior to the deacylation half reaction.

**Table 3:** Experimental and Calculated Kinetic Parameters for the SARS-CoV-2 3CL Protease^a^

proposed rate constant	*k*_1_ (M^–1^ s^–1^)	*k*_2_ (s^–1^)	*k*_3_ (s^–1^)	*k*_4_ (s^–1^)	*k*_5_ (s^–1^)	*k*_7_ (s^–1^)	*k*_9_ (s^–1^)	*k*_10_ (s^–1^)	*k*_11_ (s^–1^)	*k*_13_ (s^–1^)
	2.1 × 10^7^	200	275	450	130	5000	80	45	90	7.5

known^b^ and proposed sKIEs	^D^*K*_taut1_	^D^*k*_5_	^D^*k*_9_	^D^*k*_11_	^D^*k*_eq 9_^b^
	0.53	1.1	1.3	1.1	1.3

kinetic parameter	*k*_cat_/*K*_a_ (M^–1^ s^–1^)	*k*_ac-pss_ (s^–1^)	*k*_dac_ (s^–1^)	*k*_cat_ (s^–1^)	^D^*k*_cat_/*K*_a_	^D^*k*_ac-pss_	^D^*k*_dac_	^D^*k*_cat_
experimental	(2.6 ± 0.2) × 10^4^	42 ± 1	5.91 ± 0.03	5.2 ± 0.2	0.56 ± 0.02	0.54 ± 0.02	1.00 ± 0.02	1.04 ± 0.09
calculated	2.6 × 10^4^	42	6.13	5.0	0.56	0.58	1.05	0.98

a Experimental kinetic parameters are those reported in Tables 1 and 2. Calculated kinetic parameters were obtained by inserting the proposed rate constants and sKIEs into eqs 21–31.

b References (24 and 25).

From the mechanism in Figure 9, expressions for kinetic parameters or sKIEs are given by eqs 19–29, in which we use the leading superscript D to indicate an sKIE on a given kinetic step or parameter.
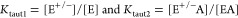
19

The fractions of the tautomeric forms in free or substrate-bound enzyme are given in eq 20.
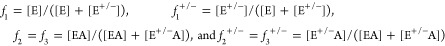
20

From eq 18, it holds that
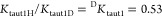
21
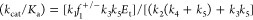
22
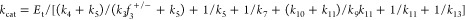
23
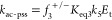
24
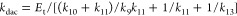
25
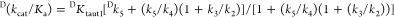
26
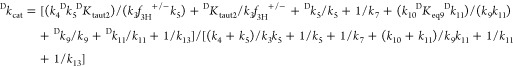
27
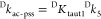
28
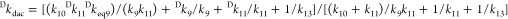
29

Values of ^D^*K*_taut1_ = ^D^*K*_taut2_ = 0.53 and ^D^*K*_eq 9_ = 1.1 were similar to those previously ascertained from evaluation of fractionation factors for identical kinetic steps for cruzain^24^ and cathepsin C.^25^

While it is not possible to simultaneously solve eqs 22–29 to obtain microscopic rate constants *k*_1_–*k*_13_ or the intrinsic kinetic isotope effects ^D^*k*_5_, ^D^*k*_9_, and ^D^*k*_11_, we selected a large range of possible values for these rate constants and intrinsic kinetic isotope effects, inserted these into eqs 22–29, and then solved the kinetic parameters and sKIEs.

The kinetic parameters and sKIEs we calculated from the proposed rate constants and sKIEs are in excellent agreement (less than 8% difference) with the experimental values, as shown in Table 3. This in no way means that these are unique and true values for these proposed constants, but since all of these values are self-consistent in the solution of eight separate kinetic expressions, they are reasonable estimates of the true values. For ^D_2_O^(*k*_cat_/*K*_a_) = 0.56 and for which ^D^*K*_taut_ = 0.53, substitution of ^D^*k*_5_ = 1.1 and (*k*_5_/*k*_4_)(1 + *k*_3_/*k*_2_)] = 0.69 into eq 27 provides the calculated value of ^D_2_O^(*k*_cat_/*K*_a_) = 0.56. Solowiej et al. reported a value of ^D^*k*_5_ = 1.8 for SARS-CoV 3CL-PR,^18^ while in our study, this intrinsic kinetic isotope effect is near unity. For the mechanism in Figure 9, this putative intrinsic sKIE represents proton transfer from His_41_H^+^ to the amino group in the tetrahedral intermediate in EX, accompanying C–N bond breakage, but its expression on the value of ^D^(*k*_cat_/*K*_a_) = 0.56 (eq 26) is attenuated by the parameter ^D^*K*_taut1_ = 0.53. The distance between the proton on the imidazolium form of His_41_ and the nitrogen of the scissile peptide bond was found to be 2.7 Å in a crystal structure of the C145A mutant of 3CL-PR to which the substrate Ac-SAVLQSGF-NH_2_ is bound.^48^ A proton transfer of this distance is likely to occur in a late transition state that would be compatible with a small intrinsic sKIE of 1.1. Likewise, ^D^*K*_taut1_ suppresses the impact of the normal intrinsic sKIE ^D^*k*_5_ on the measured value of ^D^*k*_ac_ = 0.54. The near identity of values of *k*_cat_ and *k*_dac_ and ^D^*k*_cat_ and ^D^*k*_dac_, as well as a higher rate constant of acylation vs deacylation (*k*_ac_/*k*_dac_ = 7), indicates that the deacylation half-reaction is rate-limiting for the overall mechanism, as was observed for SARS-CoV 3CL-PR,^18^ cruzain,^24^ and cathepsin C.^25^ The small values of sKIEs on ^D^*k*_cat_ and ^D^*k*_dac_ for which ^D^*k*_9_ = 1.3, also corroborate the finding of a slower deacylation half-reaction and also implicate small intrinsic kinetic isotope effects on the deacylation half-reaction. Here, we propose values of ^D^*k*_9_ = 1.3 and ^D^*k*_11_ = 1.1 (same as ^D^*k*_5_) for the sKIEs that accompany chemical steps of the deprotonation of the lytic water and the reprotonation of Cys_145_, respectively. The proposed small intrinsic sKIEs suggest late transition states for these two proton transfers. We attribute the rate constant for release of the carboxylic product (*k*_13_ = 7.5 s^–1^) to be the slowest forward rate constant in the overall mechanism. This small rate constant, along with the small intrinsic sKIEs of ^D^*k*_9_ and ^D^*k*_11_, explains why sKIEs were not observed for ^D^*k*_cat_ and ^D^*k*_dac_.

The observed inverse values of ^D^(*k*_cat_/*K*_a_) and ^D^*k*_ac_ arise from a 2-fold enrichment of the E^+/–^ and E^+/–^A tautomers in D_2_O. This was also observed for cruzain^24^ and human cathepsin C.^25^ Therefore, the substrate binds to the minor E^+/–^ form in H_2_O, more so in D_2_O in which it is the major form, with an increased fraction of EA^+/–^, which proceeds through catalysis.

What would the sKIEs be for the chemical mechanisms of Ramos-Guzmán et al.^29^ and Świderek and Moliner^30^ for the mechanistic pathways depicted in Figure S21 (Path C and Path B, respectively)? For both of these mechanisms, the E^+/–^ form of the protease does not bind the substrate, presumably even when this thiolate-imidazolium form is enriched in D_2_O. An expression for the sKIE on the *k*_cat_/*K*_a_ kinetic parameter for Paths B and C is shown in eq 30.
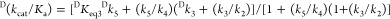
30

For both pathways, transfer of the proton from Cys_145_-SH to His_41_ proceeds via kinetic steps (*k*_3_ and *k*_4_), and not by equilibrium-driven tautomerization. One expects that ^D^*k*_3_ and ^D^*k*_4_ ≥ 1, and when ^D^*k*_3_ ∼ 2^D^*k*_4_, ^D^*K*_eq 3_ could equal ^D^*K*_taut3_ or 0.53, based on the fractionation factors described above. The sKIEs on subsequent acylation steps in both pathways will be normal; ^D^*k*_5_ ≥ 1. If ^D^*K*_eq 3_^D^*k*_5_ ≤ 0.53, ^D^(*k*_cat_/*K*_a_) = 0.56 only when *k*_5_/*k*_4_ and *k*_3_/*k*_2_ ≪ 1, that is, when the substrate is not sticky. For the mechanism of Ramos-Guzmán et al. (Path C, Figure S21),^29^ the slowest step in the acylation half-reaction is the conversion of E^+/–^A = > FP, suggesting that *k*_5_/*k*_4_ < 1. Under these conditions, ^D^(*k*_cat_/*K*_a_) could equal 0.56, as we observed. For the mechanism of Świderek and Moliner (Path B, Figure S21),^30^ the rate-limiting step in the acylation half-reaction is the conversion of EA to EX, for which equilibrium in terms of *K*_eq 3_ will not be achieved so that we may write eq 30 as eq 31.
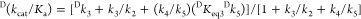
31Here, ^D^*K*_eq 3_^D^*k*_5_ can also equal 0.53–0.56 wherein ^D^*K*_eq 3_ = 0.53 and ^D^*k*_5_ = 1.0–1.1, and ^D^*k*_3_ ≥ 1 as discussed above. The *k*_3_/*k*_2_ term will be much less than 1, while the value of *k*_4_/*k*_5_ is likely to be less than 1 since *k*_5_ > *k*_3_, *k*_4_. However, a value of ^D^(*k*_cat_/*K*_a_) ∼ 0.56 can only be achieved when ^D^*k*_5_ = 1.0, ^D^*k*_3_ = 1, *k*_3_/*k*_2_ = 0–0.3, and *k*_4_/*k*_5_ = 15, for which this value of *k*_4_/*k*_5_ is unlikely. While inverse sKIEs for 3CL-PR could be observed for Paths B and C based on the conditions set forth above, all of this is predicated on the absence of binding of the substrate to the E^+/–^ form. Neutron diffraction data^52^ of the apo form of 3CL-PR unequivocally demonstrated that this is the major enzyme form in D_2_O in which the value of *k*_cat_/*K*_a_ is twice this value in H_2_O. This is the best evidence that the substrate used in our studies binds to the E^+/–^ enzyme form in both solvents.

As the pH-inhibition profile of the peptide aldehyde inhibitor BC-666 is bell-shaped and is effectively superimposable on the substrate plot of log(*k*_cat_/*K*_a_) vs pH, one may conclude that this potent, time-dependent inhibitor also binds to the minor fraction E^+/–^ to form the thiohemiacetal adduct without the need for deprotonation of Cys_145_-SH. Such an equilibrium-driven conversion of the predominant neutral E to form to E^+/–^ may account for the lengthy time-dependent inhibition observed for this inhibitor as well as nirmatrelvir and other electrophilic inhibitors of 3CL-PR, which also “select” the thiolate in E^+/–^. An inhibitor of 3CL-PR that binds to the neutral E form, which then effects deprotonation of Cys_145_-SH to produce Cys_145_-S^–^, followed by adduct formation with the resulting thiolate, may provide a new class of inhibitors that exert their full potency at earlier timeframes. Such inhibitors would also be more selective, as their action requires a proton transfer in the active site of 3CL-PR, and not other cysteine proteases.

## Data Availability

All data supporting the findings in this study are available within the paper and its Supporting Information files.

## Supporting Information Available

Scheme for synthesis of, proton NMR spectra of, HPLC of BC-666; HPLC and mass spectra of Abz-Ser-Ala-Val-Leu-Gln-Ser-Gly-Phe-Arg-Lys(Dnp)-NH_2_; purification scheme of SARS-CoV-2 3CL-PR; SDS-PAGE results of HisTrap purification and GSTrap purification after HRV-3CL-PR cleavage of SARS-CoV-2 3CL-PR; SDS-PAGE result of gel filtration purification for SARS-CoV-2 3CL-PR after anion exchange purification; standard curve of gel filtration column; purification of HRV-3C protease; initial velocity data of two FRET-based peptide substrates of 3CL-PR; pseudo-first-order inactivation of 3CL-PR; replots of values of steady state and transient rate vs individual substrate concentrations in H_2_O and D_2_O; pre-steady-state time courses of fluorescence data; and additional data on materials and methods (PDF)
