# Supplementary material for: Catalytic Mechanism of SARS-CoV-2 3-Chymotrypsin-Like Protease as Determined by Steady-State and Pre-Steady-State Kinetics
Source: ACS Catal. 2024 Nov 27;14(24):18292–309. doi: 10.1021/acscatal.4c04695 (PMC11667672; doi:10.1021/acscatal.4c04695)
Supplement: Supplementary file 1 — cs4c04695_si_001.pdf [file cs4c04695_si_001.pdf]

SUPPORTING INFORMATION

Catalytic Mechanism of 3-Chymotrypsin-Like Protease from SARS-CoV-2 as Determined from Solvent Kinetic Isotope Effects of Steady-State and Pre-Steady-State Kinetics[‡]

Jiyun Zhu[§,1], Alexandria M. Kemp,[1] Bala C. Chenna,[1] Vivek Kumar,[1] Andrew Rademacher,[1] Sangho Yun,[2] Arthur Laganowsky,[2] and Thomas D. Meek[1,2]*

Departments of Biochemistry and Biophysics[1] and Chemistry[2], Texas A&M University, College Station, Texas, 77843

[§]300 Pasteur Drive, Edwards Building, Department of Pathology, Stanford University. Stanford, CA, 94305− 5324

*Author to whom correspondence should be addressed

Phone: (979) 458 9787

Email: Thomas.Meek@ag.tamu.edu

Figures……………………………………………………………………………………S2-S19

Methods…………………………………………………………………………….....S20-S30

References……….…………………………………………………………………...…S30

Abbreviations………………………………………………………………...………S31

FIGURES

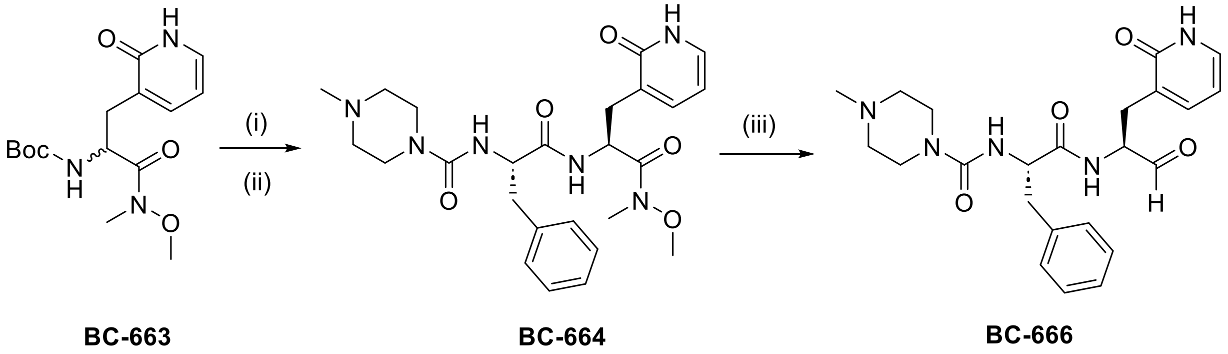

(i) TFA, DCM, 0 ºC, 2h; (ii) NMe-Pip-Phe-OH, T3P, DIPEA, DCM, -10 ºC, 10 min, 0 ºC, 1 h; (iii) LAH, THF, -10 ºC, 30 min.

Figure S1. Synthetic scheme for the synthesis of BC-666

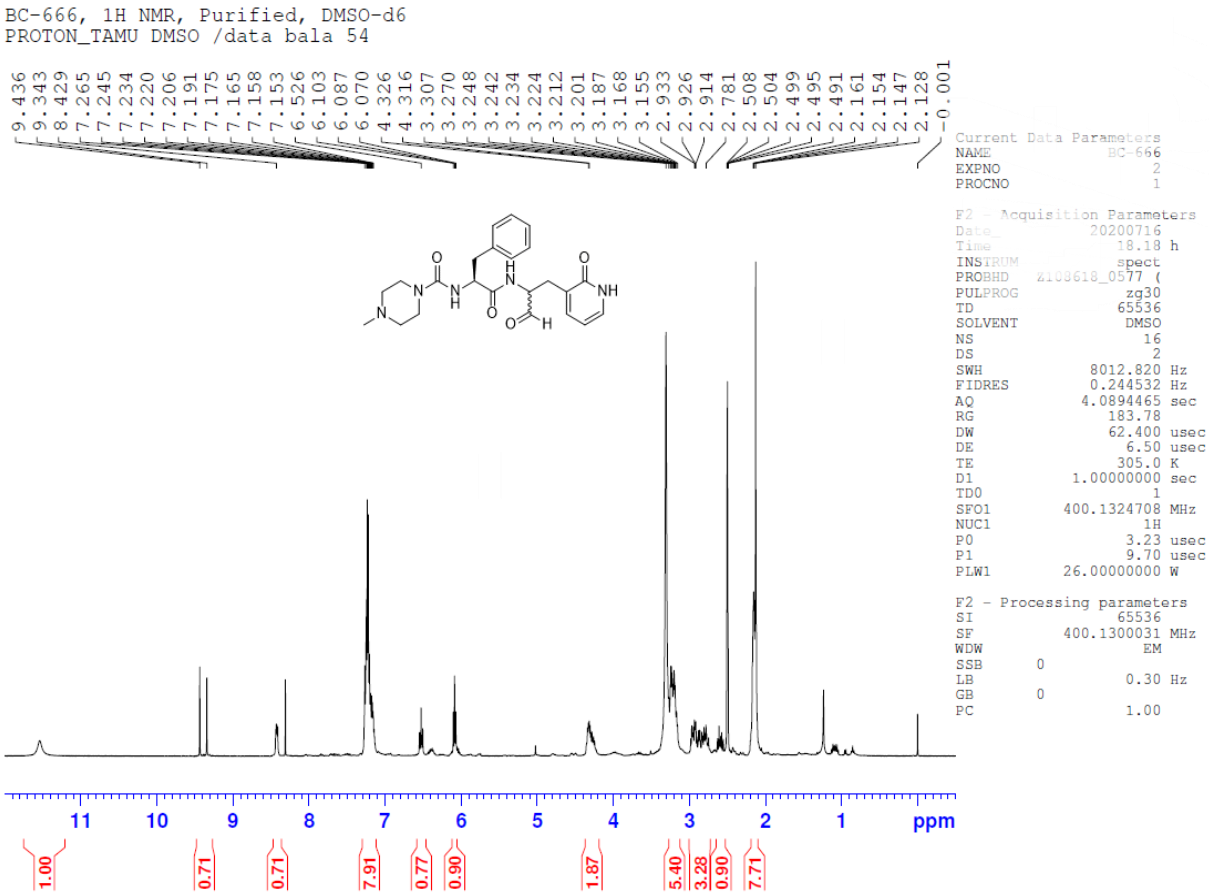

Figure S2. Proton NMR spectra of BC-666.

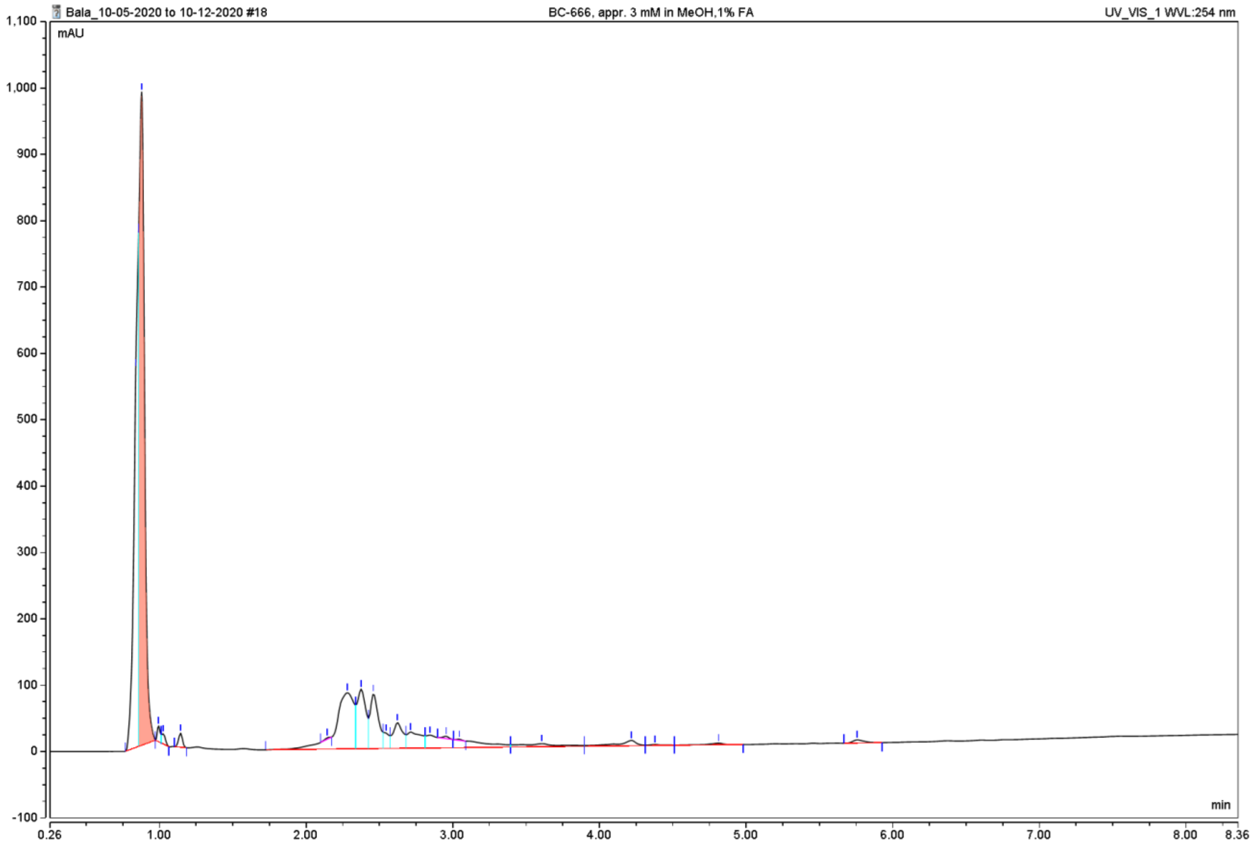

Figure S3. High performance liquid chromatography of **BC-666** as described in Materials and Methods.

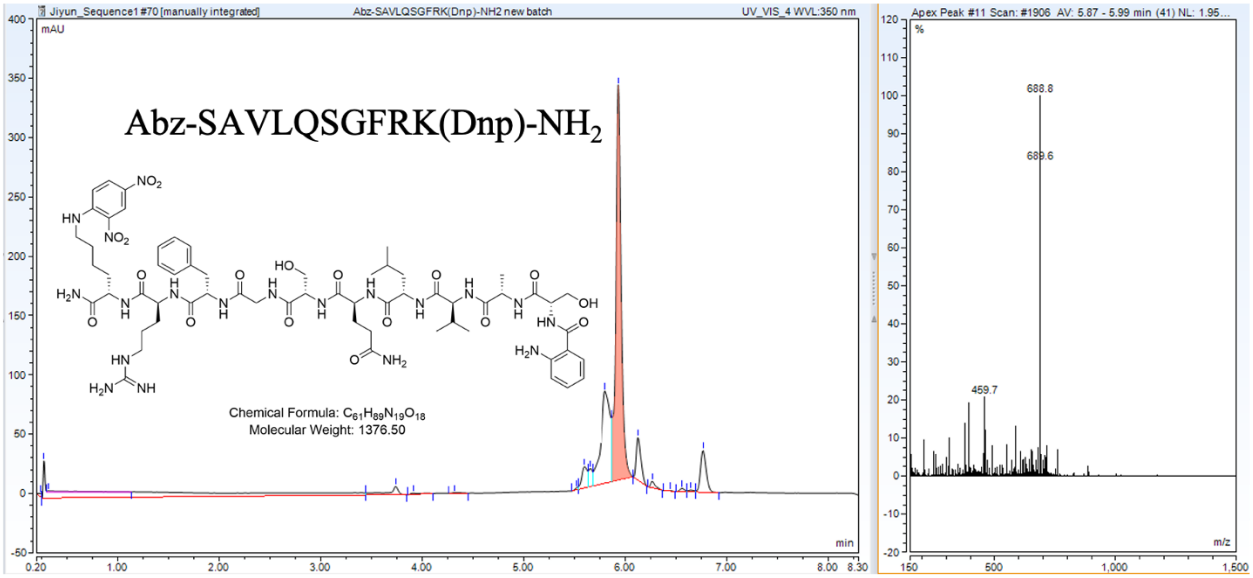

Figure S4. High performance liquid chromatography and mass spectra of synthetic substrate Abz-Abz-Ser-Ala-Val-Leu-Gln-Ser-Gly-Phe-Arg-Lys(Dnp)-NH_2, as described in Materials and Methods.

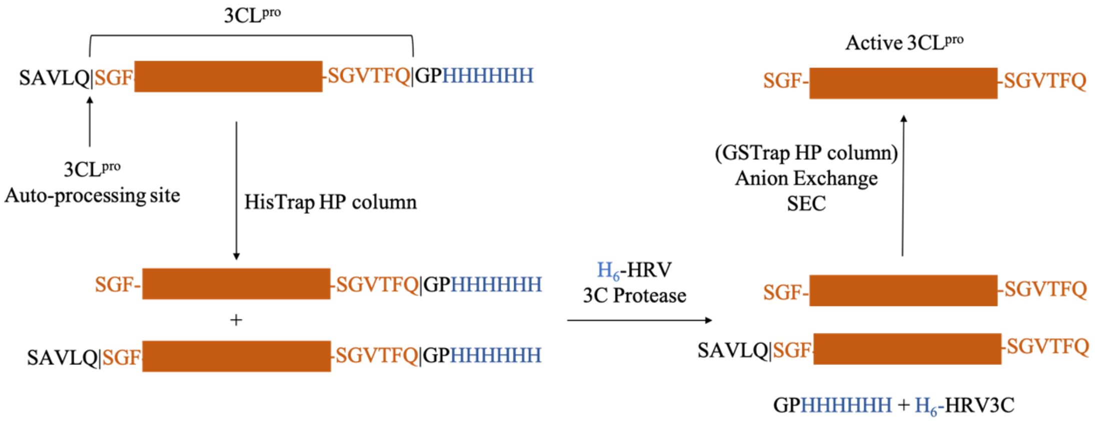

Figure S5. Purification scheme of SARS-CoV-2 3CL-PR. After cell lysis, SARS-CoV-2 3CL-PR is first enriched by HisTrap column, and then the H6-HRV-3C protease will cleave the C-terminal tag. Anion exchange and gel filtration helps remove H_6-HRV-3C protease for the production of pure and active SARS-CoV-2 3CL-PR.

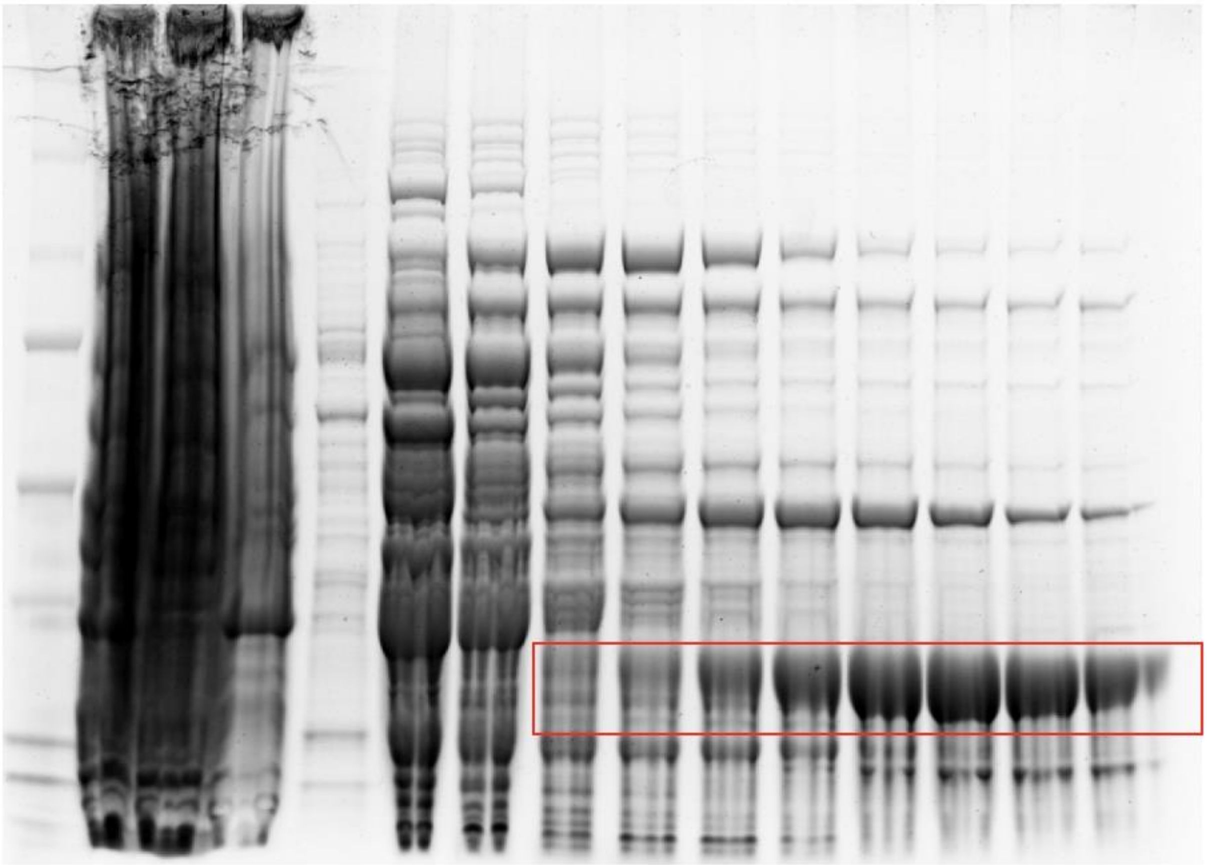

Figure S6. First HisTrap purification of SARS-CoV-2 3CL-PR from Cnstr1. Lane 1: Bio-Rad all blue ladder. Molecular weight markers are from top to bottom: 250, 150, 100, 75, 50, 37, 25, 10, 15 and 10 kDa. Lane 2: whole cell lysate. Lane 3: cell debris. Lane 4: supernatant. Lane 5: flow-through. Lane 6 and 7: fractions collected from washing step. Lane 8-15: fraction collected from the elution step. The bands of 3CL-PR are highlighted in the red box.

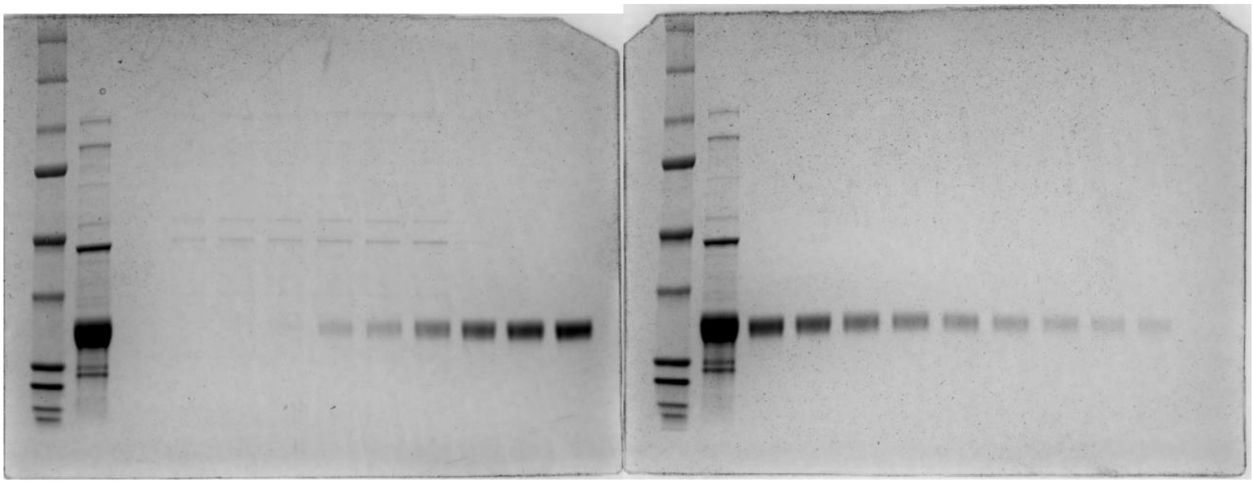

Figure S7. SDS-PAGE result of HisTrap purification (left panel) and GSTrap purification(right panel) after HRV-3CL-PR cleavage of SARS-CoV-2 3CL-PR(Cnstr1). Left panel: Lane 1: Bio-Rad all blue ladder, Lane 2: Pooled fraction after HRV-3CL-PR cleavage, Lane 3-6: fractions from washing step. Lane 7-12: fractions from elution step. Right panel: Lane 1: Bio-Rad all blue ladder, Lane 2: Pooled fraction after HRV-3CL-PR cleavage, Lane 3-12: fractions collected from the washing step. Molecular weight markers are from top to bottom: 250, 150, 100, 75, 50, 37, 25, 10, 15 and 10 kDa.

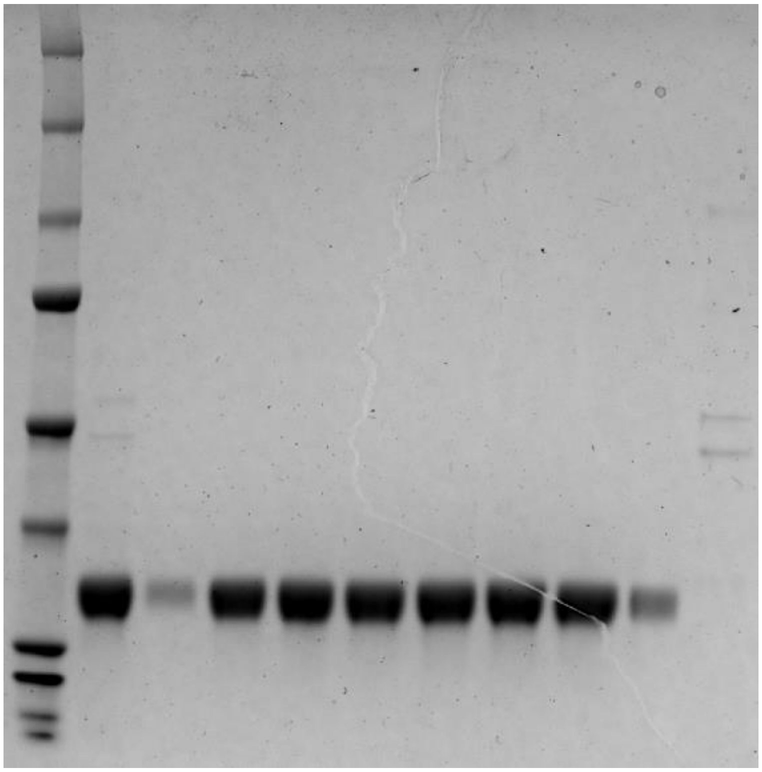

Figure S8. SDS-PAGE result of anion exchange purification after first HisTrap purification of 3CL-PR (Cnstr1) and HRC-3C-PR cleavage. Lane 1: Bio-Rad all blue protein standards ladder. Lane 2: Pooled fraction after 2nd HisTrap. Lane 3-10: flow-through collected. Molecular weight markers are from top to bottom: 250, 150, 100, 75, 50, 37, 25, 10, 15 and 10 kDa.

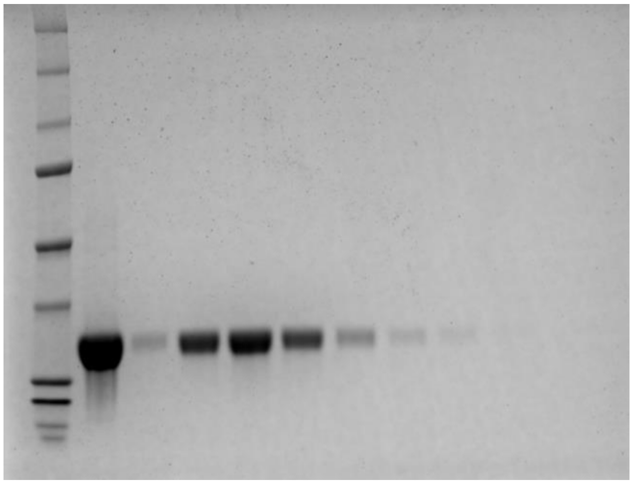

Figure S9. SDS-PAGE result of gel filtration purification for SARS-CoV-2 3CL-PR(Cnstr1) after anion exchange step. Lane 1: Bio-Rad all blue ladder, Lane 2: Pooled and concentrated fraction after anion exchange purification, Lane 3-10: fractions collected from the size-exclusion purification column. Molecular weight markers are from top to bottom: 250, 150, 100, 75, 50, 37, 25, 10, 15 and 10 kDa.

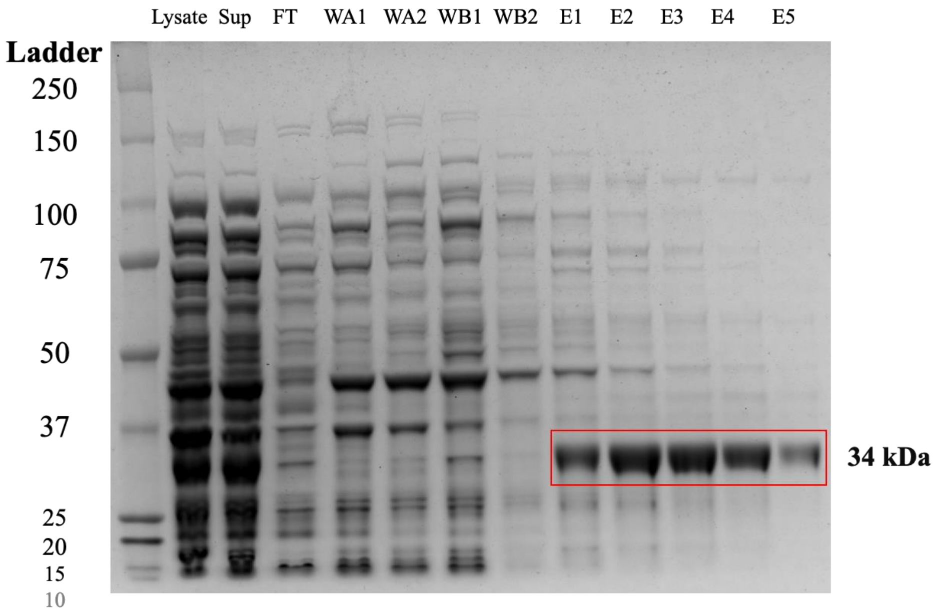

Figure S10. First HisTrap purification of SARS-CoV-2 3CL-PR from Cnstr2. Sup: supernatant. FT: flow-through. WA1: washing buffer A 1st round. WB2: washing buffer B second round. E: elution.

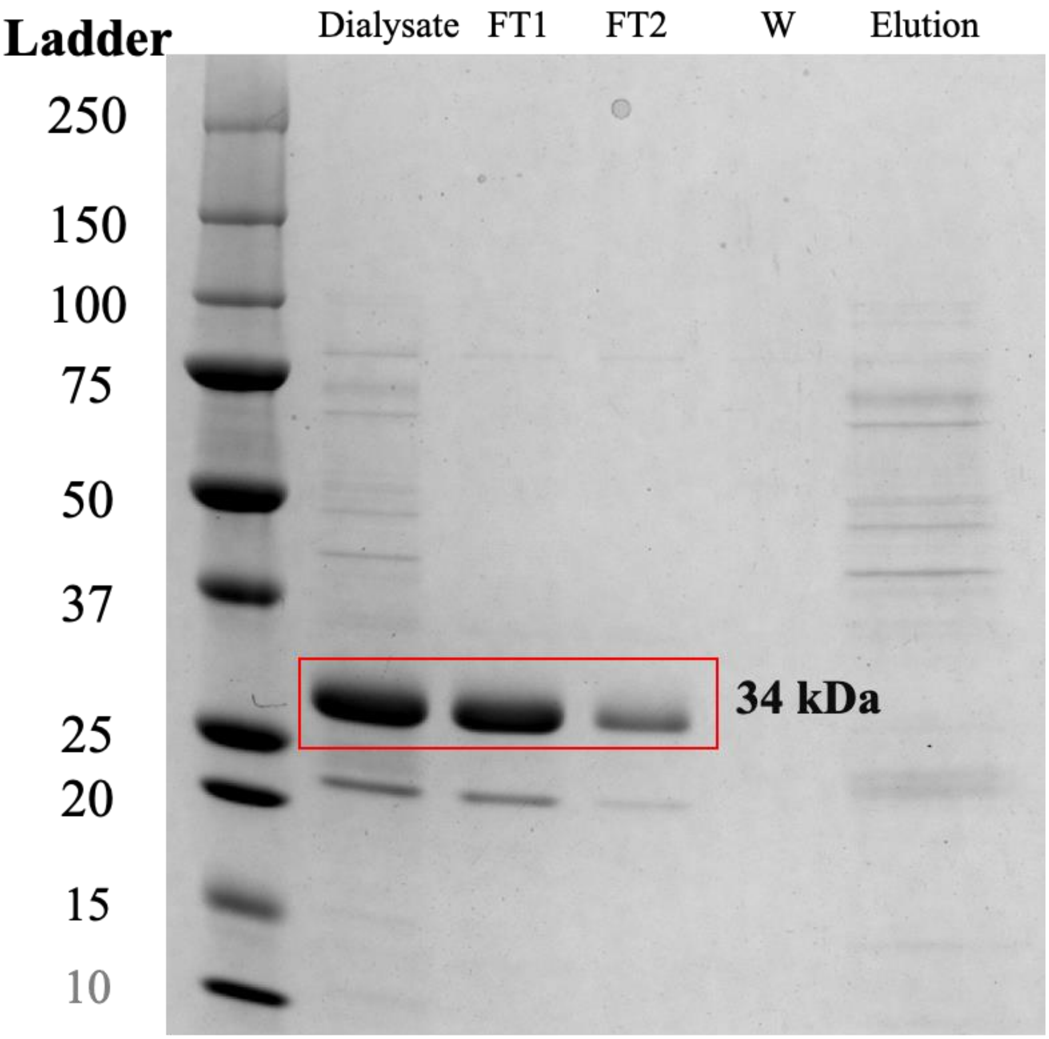

Figure S11. Anion Exchange purification of SARS-CoV-2 3CL-PR (Cnstr2). FT: flow-through. W: sample collected from washing step.

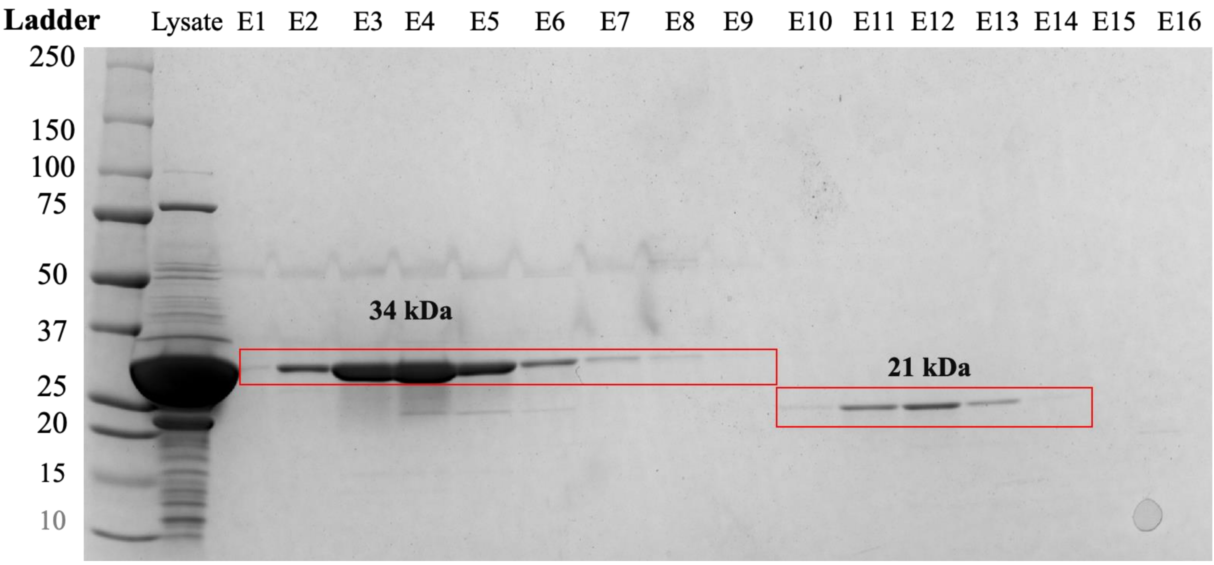

Figure S12. Gel filtration purification of SARS-CoV-2 3CL-PR (Cnstr2). E: elution. Numbers after E refers to the tube number of collected sample. 21-kDa bands are from the H6-HRV-3C protease used for 3CLpro cleavage.

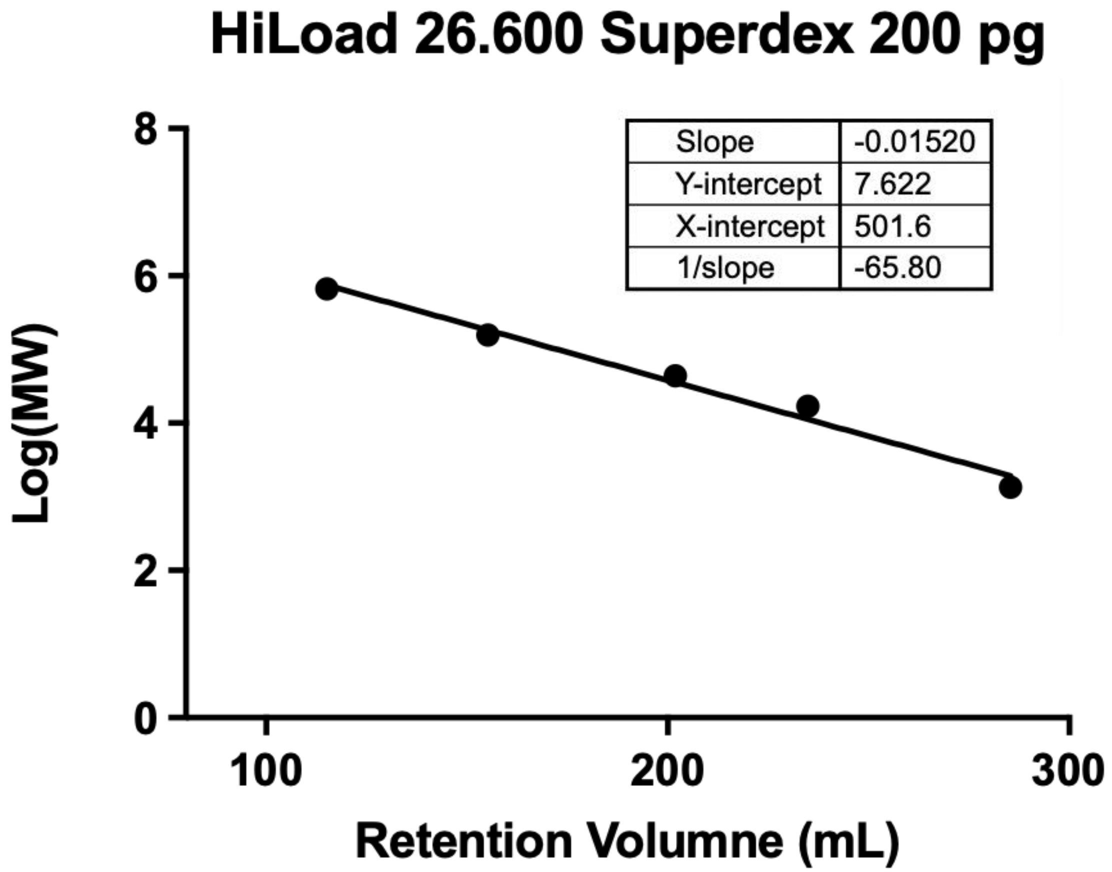

Figure S13. Standard curve of gel filtration column. The elution volume of protein is 183 mL, between the elution volume of γ-globulin (bovine, 158 kDa) and Ovalbumin (chicken, 44 kDa), (manufacturer: BioRad chrome-extension://efaidnbmnnnibpcajpcglclefindmkaj/https://www.bio-rad.com/webroot/web/pdf/lsr/literature/MSLIT-102E.pdf).

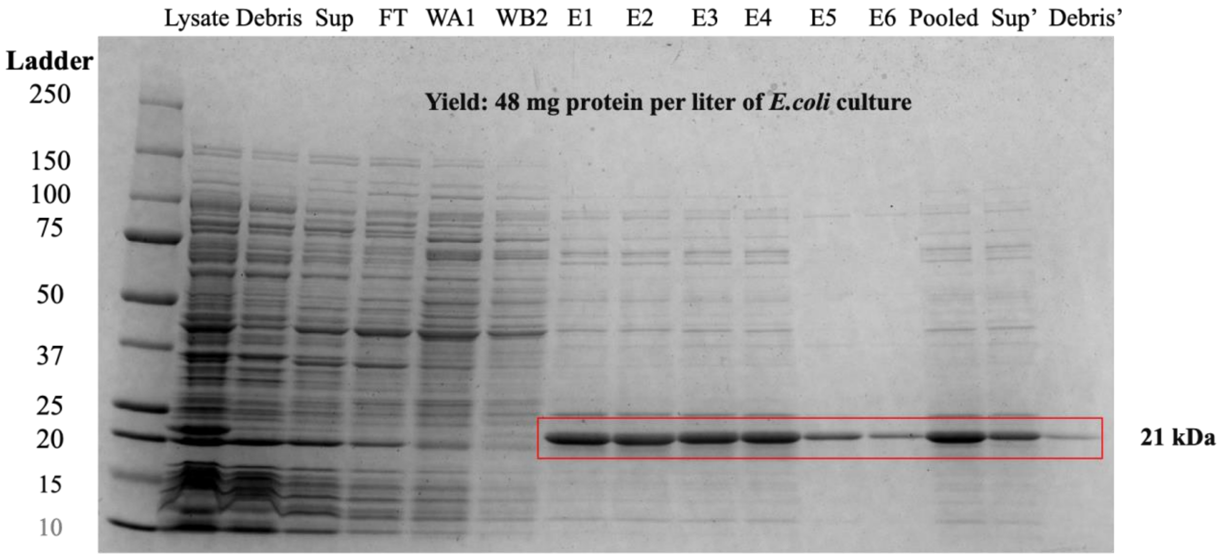

Figure S14. Purification of HRV-3C protease. Sup: supernatant. FT: flow-through. WA1: washing buffer A 1st round. WB2: washing buffer B 2nd round. E: elution fractions. Sup': supernatant of pooled fractions. Debris: precipitation from dialysis.

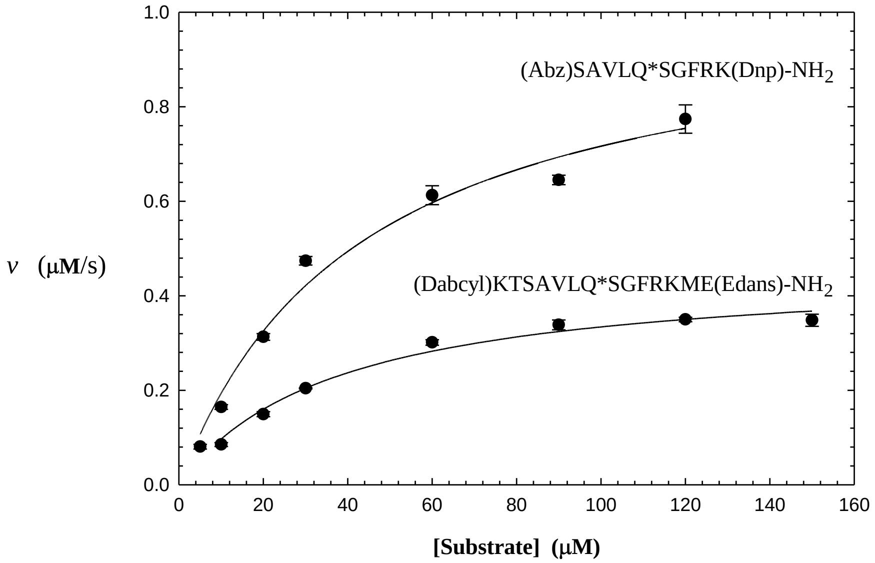

Figure S15. Initial velocity data of two FRET-based peptide substrates of 3CL-PR acquired at pH 7.5 in Assay Buffer with 0.5 mg/mL BSA. The lines drawn through the experimental data were from fitting of data to eq 1.

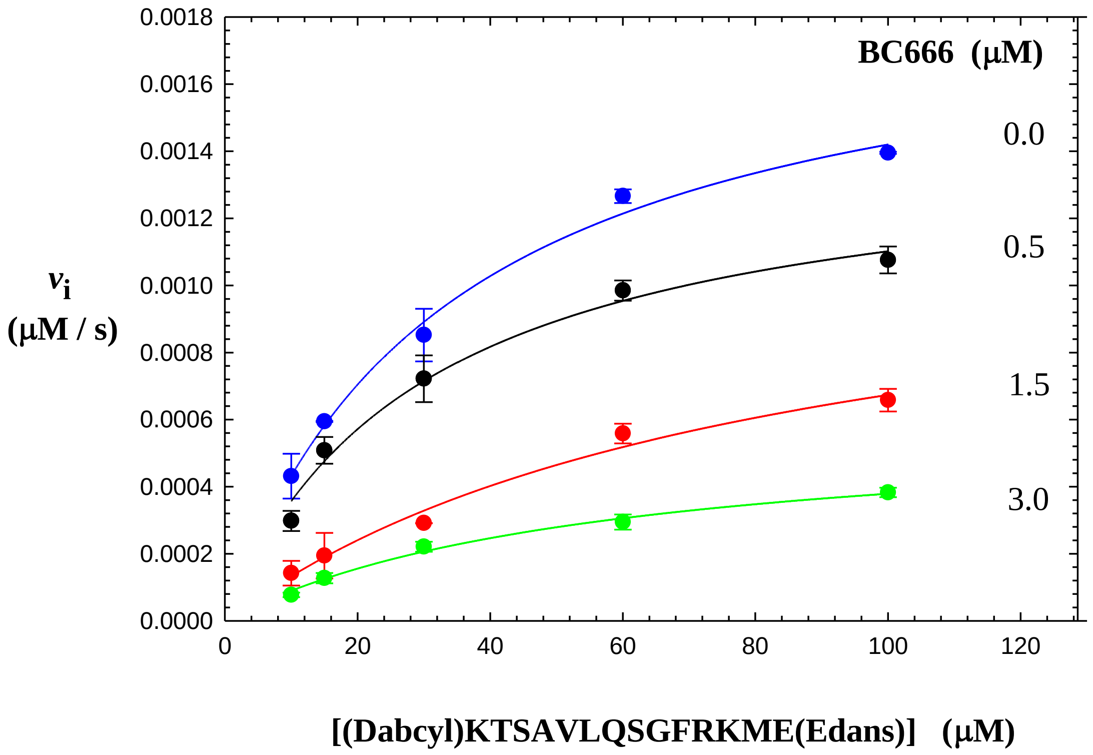

Figure S16. Plot of initial rate data (0-3 min after addition of 4.8 nM 3CL-PR) vs. [(Dabcyl)-KTSAVLQSGFRKME(Edans)] (μM; 15-75 μM) at 0-3 μM BC-666. Initial rate were acquired after addition of 3CL protease to reaction mixtures at 0-3 min. Data were fitted to eqs 2 and 3, competitive and non-competitive inhibition, respectively, with best fitting to eq 2, $\boldsymbol{V}_{max}$ = 0.0018 ± 0.0001 μM/s, $\boldsymbol{K}_m$ = 27 ± 6 μM. $\boldsymbol{K}_i$ = 0.38 ± 0.07 μM.

.

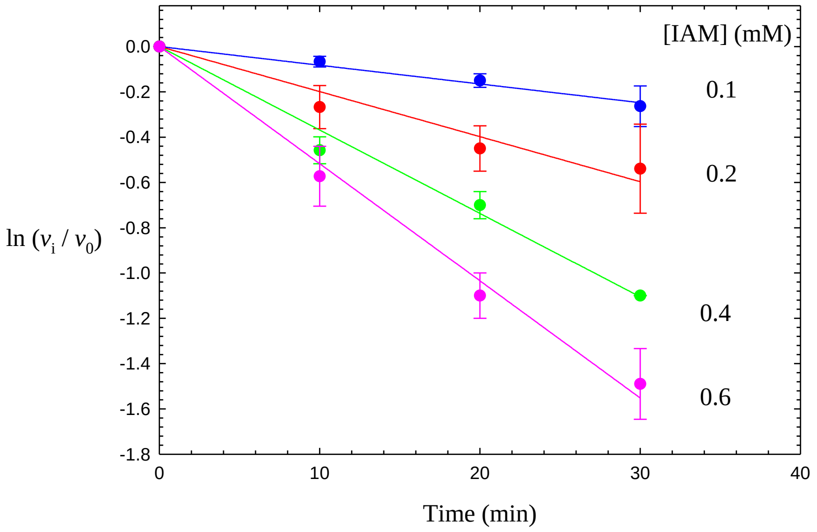

Figure S17. Pseudo-first-order inactivation of 3CL-PR by iodoacetamide (IAM) at a single value of pH 7.5. Left: Kitz-Wilson plot of the natural log of remaining 3CL-PR activity, following preincubation with multiple concentrations of IAM (average values and errors from two replicates). Each set of datapoints are color-coded according to the IAM concentration of the set. Data at each concentration of IAM were fitted using eq 10, from which the fitted lines through the experimental data points are shown.

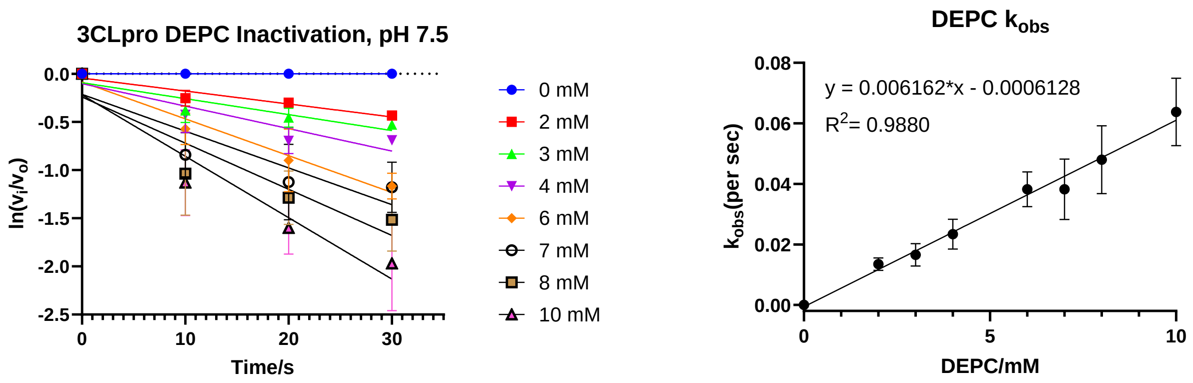

Figure S18. Pseudo-first-order inactivation of 3CL-PR by diethylpyrocarbonate (DEPC) at a single value of pH 7.5. Left: Kitz-Wilson plot of the natural log of remaining 3CL-PR activity, following preincubation with multiple concentrations of DEPC (average values and errors of two replicates). Each set of datapoints are color-coded according to the DEPC concentration of the set. Data at each concentration of DEPC were fitted using eq 10, from which the fitted lines through the experimental data points are shown. Right: Values of k_{obs} from fitting to eq 10 are replotted vs. [DEPC] with the linear parameters shown in the figure.

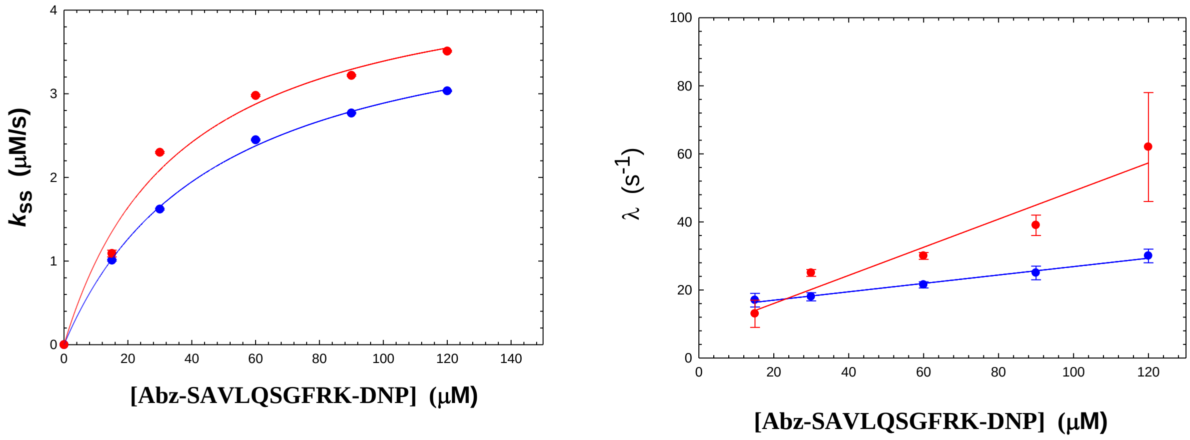

Figure S19. Re-plots of values of the steady-state (k_{ss}) and transient rates (λ) vs. individual substrate concentrations in H_2O (blue) and D_2O (red) with standard deviations shown as error bars. Fitting of the plots of k_{ss} vs. time to eq 15 gave values of: k_{catH2O} = 4.2 ± 0.1 s^{-1}, K_{aH2O} = 47 ± 3 µM, k_{catD2O} = 4.6 ± 0.4 s^{-1}, K_{aD2O} = 36 ± 8 µM s^{-1}, and fitting of the plot of λ vs. t in H_2O to eq 17 gave values of: k_{3H2O} = 28 ± 5 s^{-1}, k_{5H2O} = 6 ± 3 s^{-1}, K_{iaH2O} = 40 ± 20 mM, while fitting of the plot of λ vs. t in D_2O resulted in indeterminate values.

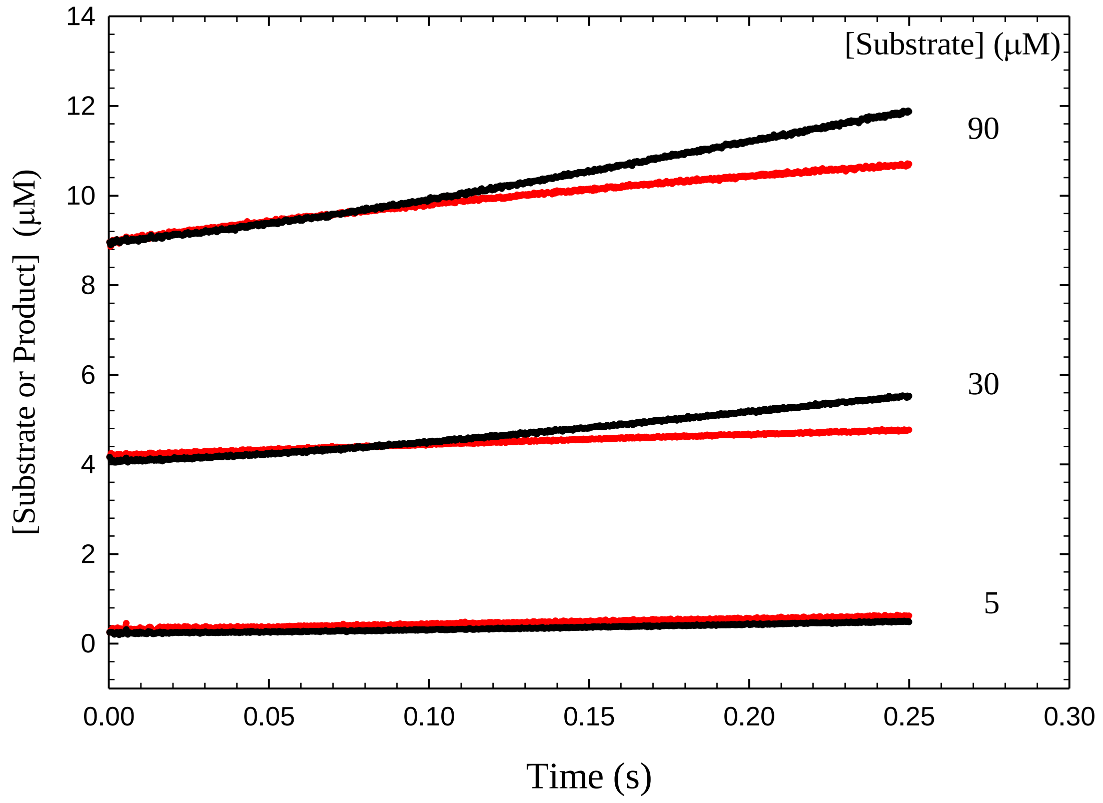

Figure S20. (**A**) Pre-steady-state time courses of fluorescence data produced by mixing of wild-type (black) or the C145A mutant (red) 3CL-PR with 5, 30, and 60 μM (Dabcyl)KTSAVLQSGFRKME(Edans)-NH_2 at 0.002-0.2 s. Kinetic parameters were obtained by fitting data at each substrate concentration to eq 12.

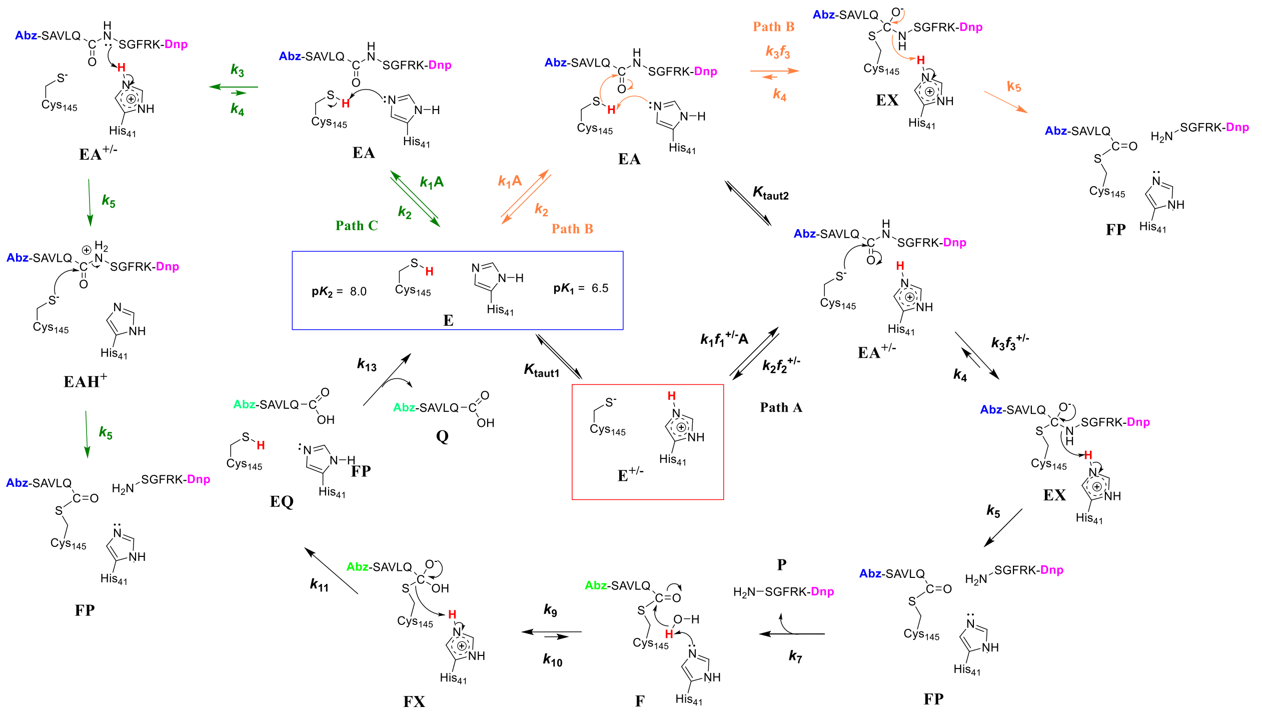

Figure S21. Modification of **Fig. 9** for which the proposed mechanisms of Świderek and Moliner[30] (**Path B**) and Ramos-Guzman[29] (**Path C**) are included.

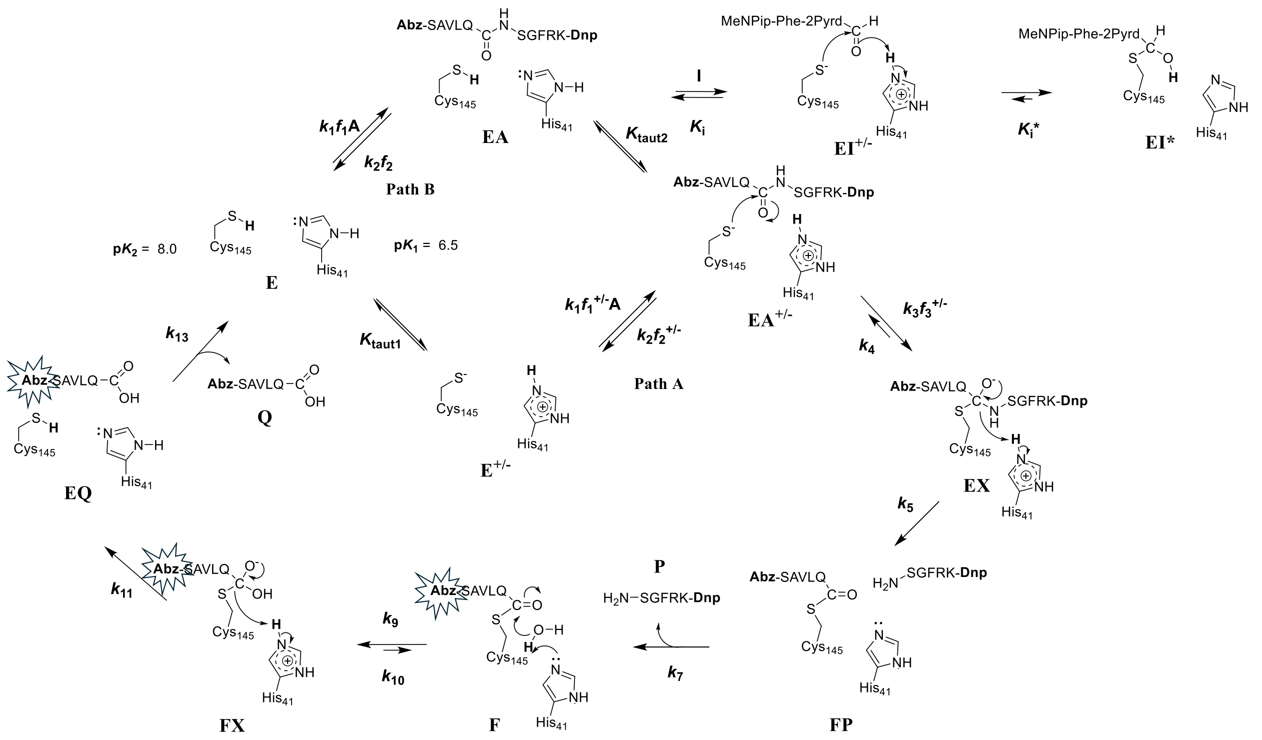

Figure S22. Black and white version of **Fig. 9**, also including which also includes a scheme for the binding of peptide aldehyde inhibitor **BC-666.** Protons expected to contribute to primary sKIEs are in bolded. The kinetic parameters for k_{cat}/K_a include the acylation kinetic steps k_1-k_7, while for k_{cat}, the de-acylation kinetic steps k_9-k_{13}. In **Path A**, defined by $\boldsymbol{k_1 f_1^{+/-}}$, substrate **A** only binds to the imidazolium-thiolate form of 3CL-PR (**E**$^{+/-}$), while in the **Path B** pathway defined by $\boldsymbol{k_1 f_1}$, **A** binds to the imidazole-thiol tautomer (**E**).

MATERIALS AND METHODS

Synthesis of BC-666. Reagents and starting materials from Commercial vendors were purchased and used as received without any purification. Reactions were carried in an inert atmosphere of nitrogen unless otherwise specified. Progress of the reactions were monitored using Thin Layer Chromatography (TLC) and LC-MS analysis, by employing an HPLC-MS (UltiMate 3000 equipped with a diode array coupled to a MSQ Plus Single Quadrupole Mass Spectrometer,

ThermoFisher Scientific) using electrospray positive and negative ionization detectors. HPLC conditions used: column: Phenomenex Luna 5 μm C18(2) 100 Å, 4.6 mm, 50 mm, Mobile phase A: water with 0.1% formic acid (v/v). Mobile phase B: MeCN with 0.1% formic acid (v/v). Temperature: 25 °C. Gradient: 0−100% B over 6 min, then a 2 min hold at 100% B. Flow: 1 mL/min. Detection: MS and UV at 254 nm.

Proton NMR spectra were obtained in DMSO-d_6 at 400MHz at 298 K on a Bruker Avance III NanoBay console with an Ascend magnet. The following abbreviations were utilized to describe peak patterns when appropriate: br = broad, s = singlet, d = doublet, q = quartet, t = triplet, and m = multiplet. The compound used for testing in assays and biological studies had purities that were determined to be >95% as evaluated by their proton NMR spectra and their HPLC/MS based on ultraviolet detection at 254 nm.

The starting compound **BC-663** (**Fig. S1**) was synthesized as per our previously reported procedure in Li *et al*. (2021) *J. Med. Chem*. 64, 11267–11287; Boc-deprotection of **BC-663** was carried using TFA and DCM. Further the product obtained was subjected to amide coupling reaction with Nme-Pip-Phe-OH (**BC-544-4**) using T3P, DIPEA in DCM, which resulted in the compound **BC-664**. LAH reduction of the Weinreb amide BC-664 yielded the target compound **BC-666**.

4-Methyl-N-((S)-1-oxo-1-(((S)-1-oxo-3-(2-oxo-1,2-dihydropyridin-3-yl)propan-2-yl)amino)-3-phenylpropan-2-yl)piperazine-1-carboxamide (***BC-666***).

The starting material **BC-664** was coevaporated with diethyl ether (3X) and dried thoroughly on high vacuum. To a solution of **BC-664** (0.051 g, 0.102 mmol) in anh. THF under a N_2 atmosphere at -10 ^{o}C was added dropwise LAH (2.0 M in THF, 0.154 mL, 0.307 mmol). The resultant reaction mixture was stirred for an additional 30 min at the same temperature. TLC (MeOH/DCM = 1:10, v/v) and LC-MS analysis revealed completion of reaction. The reaction was quenched by careful addition of EtOAc (2 mL) and then sat. aq. $NaHCO_3$ (0.5 mL) was added, followed by transfer to a separatory funnel. The contents were diluted in the funnel with EtOAc (25 mL) and sat. aq. $NaHCO_3$ (10 mL). The organic layer was separated, and the aqueous layerayer was extracted with EtOAc (2 X 25 mL). The combined organic layers were washed with brine solution (1 X 20 mL). The organic later was further dried on anh. Na_2SO_4. Thehe

drying agent was filtered, and the filtrate was concentrated on rotary evaporator. The crude product was purified using a silica gel column chromatography [MeOH/DCM, gradient 1:100 to 1:10] yielded the pure product 4-methyl-N-((S)-1-oxo-1-(((S)-1-oxo-3-(2-oxo-1,2-dihydropyridin-3-yl)propan-2-yl)amino)-3-phenylpropan-2-yl)piperazine-1-carboxamide (**BC-666**, White solid, 0.013 g, 0.030 mmol, 29% yield). ^{1}H NMR (400 MHz, DMSO-d_6) δ 2.09 – 2.19 (m, 7H), 2.53 – 2.65 (m, 1H), 2.73 – 3.01 (m, 3H), 3.12 – 3.28 (m, 5H), 4.19 – 4.37 (m, 2H), 6.09 (t, 1H, *J* = 6.6 Hz), 6.53 (t, 1H, *J* = 8.3 Hz), 7.11 – 7.30 (m, 8H), 8.42 (q, 1H, *J* = 3.4 Hz), 9.29 – 9.48 (m, 1H), 11.5 (bs, 1H); (**Fig. S2**) LC-MS *m/z* 440.2, 441.3 $[M+H]^+$, ($C_{23}H_{29}N_5O_4^+$ Calcd 440.23); t_R = 0.89 min (**Fig. S3**).

Synthesis of N-amino-benzoyl-Ser-Ala-Val-Leu-Gln-Ser-Gly-Phe-Arg-Lys-(ε-2,4-dinitro-benzyl)-NH$_2$.

The synthesis of (Abz)SAVLQSGFRK(Dnp)-NH$_2$ substrate using solid-phase peptide synthesis (SPPS) was conducted as described.[1] Briefly, Fmoc-protected amino acids and Rink-amide resin were used for peptide conjugation through a whole reaction cycle for each amino acid. In each cycle, the Rink-amide resin (or the Rink-amide resin attached with Fmoc-amino acid) was deprotected by treatment with 20%(v/v) N-methyl-piperidine in DMF for 45 minutes, and the presence of a free primary amine group was verified using a ninhydrin test. The resin was washed three times with DMF to remove any residual N-methyl-piperidine, followed by incubation with 1.5 eq of protected amino acid, 1.5 eq of COMU dissolved in DMF, and activated by 3 eq of DIPEA to initiate the reaction. Reaction progress was monitored by a ninhydrin test until the resin bead no longer yielded blue color. To block unprotected primary amines, samples were incubated with activated 25% (v/v) acetic anhydride in DMF for 30 minutes. Then the resin was washed with DMF for the next round of reaction until the final coupling of protected serine at the N-terminus was completed. Fmoc-SAVLQSGFRK(Dnp)-NH$_2$ was cleaved from the Fmoc-SAVLQSGFRK(Dnp)-resin, and analysis of the resulting peptide by LC-MS provided confirmation of its sequence, prior to the last coupling reaction of Boc-2-Abz-OH to Fmoc-SAVLQSGFRK(Dnp)-resin, which was followed by washing once with DMF and three times by DCM. The resin was either stored in a desiccator at ambient temperature or resuspended in a cleavage solution containing 95% (v/v) TFA, 2.5% (v/v) water, and 2.5% (v/v) TIPS before purification. For product extraction, the peptide crude product was precipitated by evaporating the

TFA under nitrogen gas flow, followed by the addition of ice-cold diethyl ether, centrifugation at 4ºC, separation, and drying of the precipitant. The dried crude product was either stored at -20 ºC or resuspended in 2 mL of DMF solution with the addition of 100 μL of DMSO until full dissolution was achieved. Solutions of the peptide in DMF were purified by semi-preparative HPLC using a Thermo Fisher Scientific UltiMate 3000 equipped with a column of Phenomenex Luna 5 μm C18(2) 100 Å, 21.2 mm, 250 mm, with a single-wavelength detector coupled to a fraction collector. Purifications were conducted using the following conditions: elution at 21.2 ml/min using 98% water/2% acetonitrile with 0.1% formic acid (A) and 2% water/98% acetonitrile with 0.1% formic acid (B) as mobile phase (**Fig. S4**). The gradient used was 5%-52% B over 20 mins, then 52-100% B to 26 minutes, and 100% B until 29 minutes at 21.2 ml/min). The fractions containing pure peptide (≥ 95% pure) were then lyophilized to yield a fluffy yellow powder (42.4 mg, 29.2% yield as a formic acid salt) LC-MS t_R: 5.36 min, m/z 688.89 [M+2H], 459.79 [M+3H], $C_{61}H_{89}N_{19}O_{18}$ Calcd 1376.66 [M+1H], 688.83 [M+2H], 459.56 [M+3H].

Elution fractions containing the desired product were pooled, concentrated by use of a SpeedVac , and then lyophilized for 2 days to remove water in the product powder. For assays, the powder was dissolved into 100% DMSO stock solutions stored in all-black Eppendorf tubes under -20 ºC, and thawed at ambient room temperature before usage.

Recombinant Expression and Purification of Wild-Type and Cys145Ala SARS-CoV-2 3CL protease. We used four expression constructs to make 3CL-PR in *E. coli*; the first was GSTtag-SARS-CoV-2 3CLpro-HisTag (Cnstr1), the second was HisTag-SARS-CoV-2 3CLpro-HisTag (Cnstr2), the third was His-SUMOTag-SARS-CoV-2 3CLpro (Cnstr3) and the fourth was the His-SUMOTag-SARS-CoV-2 C145A mutant (Cnstr4). Following purification and processing, the primary sequences of all 3CL-PR were identical expect the C145A mutant. An overall purification scheme is shown in **Fig. S5** for the first two constructs. Protein purity was evaluated by SDS-PAGE which indicates protein purity (**Figure S6-S15**).

SARS-CoV-2 3CL protease (3CL-PR) Cnstr1 and Cnstr2 were expressed in *E. coli* and purified as described,[1] with minor changes to enhance the final protein yield (**Fig. S5**). All buffers were filtered through 0.2-µm filters before use, and buffer used for size-exclusion chromatography was thoroughly degassed after filtration. Briefly, the plasmid encodes a reading frame which will express a protein of the form N-term-GST-tag/SAVLQ*SGF-3CL-PR-SAVLQ*SGF/SGVTFQ*GP/His_6-tag-C-term. The N-terminal side of the 3CL-PR, coding sequence is flanked by either a GST-tag or a His_6-tag followed by the 3CL-PR cleavage sequence (SAVLQ*SGF). On the C-terminal side of the protease coding sequence, there is a modified HRV-3C protease cleavage-site sequence (SGVTFQ*GP) preceding a His_6-tag to prevent auto-proteolysis of 3CL-PR after expression. 10-mL of LB media containing 100 µg/mL carbenicillin was inoculated with a glycerol stock of *E.coli* at 37 ºC overnight, as a starter culture, before being transferred into 2 L of LB media containing 100 µg/mL carbenicillin for large-scale growth. The culture was chilled on ice after OD_{600} had reached 0.6, before IPTG (0.5 mM or 1 mM) was added to induce protein expression at 24 ºC or 18 ºC overnight. Bacterial cells were centrifuged at 6,300 x g, 4 ºC for 45-60 minutes, and cell pellets were immediately lysed or stored under – 20 ºC for future use.

For protein purification, cell pellets stored at -20 ºC were thawed on ice or at 4 ºC, and then resuspended in Lysis Buffer (12 mM or 20 mM Tris-HCl (pH 7.5), 120 mM or 200 mM NaCl) at a ratio of 10-mL Lysis Buffer per 1 g pellet. The resulting suspension solutions were passed three times through French Press at 25,000 psi to ensure thorough lysis. Lysates were centrifuged at 26,000 x g for 45-60 minutes (4 ºC) to remove cell debris. Each supernatant was passed through a 0.45-µm filter prior to application to a 15-mL column of nickel-NTA resin, which was then washed twice with three column volumes of a 96:4 ratio of Lysis Buffer: Elution Buffer (20 mM Tris-HCl

(pH 7.5), 200 mM NaCl, 250 mM imidazole), followed by washings with 2 column volumes (CVs) of 80:20 Lysis Buffer:Elution Buffer. Elution of resin-bound protein was achieved by repeatedly passing 1 CV of Elution Buffer through the column until the eluate solution contained no protein detectable by use of the Bradford reagent. The presence of 3CL-PR in the eluate was confirmed by the observation of a 34k-Da band upon analysis of fractions by SDS-PAGE (**Figs. S6-S12**), prior to pooling of eluates and dialysis with two exchanges of Dialysis Buffer (20 mM Tris-HCl (pH 7.5), 150 mM NaCl, 2 mM DTT) at 4 ºC for 4 hours. The cleavage of the C-terminal His-tag was initiated by adding 2 mg of His_6-HRV-3C protease into the eluates while continuing dialysis for at least 18 hours. Separation of the cleaved His tag, His_6-HRV-3C protease, as well as other proteins, from the SARS-CoV-2 3CL-PR was achieved by either loading the dialysate on to a GSTrap column, followed by a HisTrap column, or through a HiTrap anion exchange column. For SARS-CoV-2 3CL-PR, examination by SDS-PAGE (**Fig. S6**) indicated the existence of uncleaved 3CL-PR in the cell lysate, as well as in the eluant from the first HisTrap column. After HRV 3C protease-catalyzed cleavage, the activity of the 3CL-PR was confirmed by the addition of small aliquots of the dialysate (containing active SARS-CoV-2 3CL-PR) into a reaction mixture solution containing the FRET-based substrate: instant cleavage of the substrate led to a jump in the RFU intensity (data not shown) from plate-reading, suggesting the high activity of 3CL-PR in the sample. For purification using anion exchange, the sample solution was passed through a pre-equilibrated HiTrap column with 15% Buffer C (20 mM Tris-HCl (pH 7.5), 2 mM DTT) + 85% buffer D (20 mM Tris-HCl pH 7.5, 1000 mM NaCl, 2 mM DTT) (**Fig. S7**). Eluant containing purified 3CL-PR was concentrated using Amicon filtration (MWCO 10-kDa) to a concentration of ~20 mg/mL, prior to size-exclusion chromatography. For SEC purification, 1-mL samples of ~ 20 mg/mL 3CL-PR (to achieve a sample volume under 1% of that of the HiLoad (26/600) gel-

filtration column volume (120-124 mLs) pre-equilibrated using buffer C containing 150 mM NaCl. Elution with this buffer after sample application resulted in collection of fractions of SARS-CoV-2 3CL-PR as confirmed by calculating the elution volume from a standard curve based on molecular weights of protein standards, as well as confirmation of present 3CL-PR by SDS-PAGE (**Fig. S7 and S13**). Fractions containing SARS-CoV-2 3CL-PR were combined and concentrated to ~ 10 mg/mL before either flash-freezing in liquid N_2 of 50-µL aliquots for future crystallization, or storage in 20 mM Tris-HCl (pH 7.5), 150 mM NaCl, 2 mM DTT, 0.1 mM EDTA-Na_2, and 50% (v/v) glycerol at -80 °C for use in kinetic studies.

The elution volume of 3CL-PR from size-exclusion chromatography was 183 mL, between the elutions of γ-globulin (bovine, 158 kDa) and Ovalbumin (44 kDa), was consistent with 3CL-PR existing as a homodimer of two 34-kDa subunits. The catalytic activity of the protein sample before and after size-exclusion chromatography indicated that there was no significant loss of activity during the purification process. The purity of SARS-CoV-2 3CL-PR samples following SEC chromatography was confirmed by SDS-PAGE analysis, which showed a single protein band corresponding to the SARS-CoV-2 3CL-PR monomer at 34 kDa.

The overall yield of SARS-CoV-2 3CL-PR purification ranged from 0.5-5 mg protein per liter bacterial culture, depending on the construct (Cnstr1 or Cnstr2) and purification method used. For a sequence of purification steps consisting of (1) HisTrap chromatography, (2) GSTrap chromatography, (3) a second purification by HisTrap chromatography, and lastly, (4) size-exclusion column chromatograhy, the overall yield of purified 3CL-PR was at the lower end of this range, which could be the result of protein loss in the flow-through or on the column (for each chromatographic step), during multiple rounds of protein purification. Another factor could be incomplete cleavage by HRV 3C protease due to insufficient incubation time, which has been

confirmed by SDS-PAGE (data not shown), where flow-through from the GSTrap and HisTrap column did not give the desired protein concentration from a Bradford test, and a 34-kDa band was observed in the elution sample of the second HisTrap column.

SARS-CoV-2 3CL protease (3CL-PR) Cnstr3 and Cnstr4 were expressed in *E. coli* BL21 (DE3) cells. All buffers were filtered through 0.2-µm filters, and degassed overnight before use in all columns. Briefly, the plasmid encodes a reading frame which will express a protein with the N-terminal His-SUMO tag; the SUMO moiety is recognized by the *S. cerevisiae* Ulp1 protease (a gift from Prof. Pingwei Li), with a cleavage site of G*SGF-3CL-PR. A 25-mL starter culture of LB media containing 100 µg/mL ampicillin was inoculated with a glycerol stock of *E.coli* at 23 ºC overnight. Fresh media was added after overnight incubation following centrifugation at 4,000 rpm. The cells were resuspended in LB media before being transferred into 1 L of LB media containing 100 µg/mL ampicillin for large-scale growth. Once OD_{600} had reached 0.6 - 0.8, the culture was allowed to cool to room temperature before IPTG (1 mM) was added to induce protein expression at 23 ºC. Bacterial cells were centrifuged at 4,000 rpm and 4 ºC for 30 minutes. EDTA-free protease inhibitor cocktail tablets were added to cell pellets suspended in 20-40 mL Lysis Buffer (50 mM sodium phosphate (pH 8.0), 300 mM NaCl, 5 mM imidazole, and 5% (v/v) glycerol, 10 mM β-ME) and were either immediately lysed by sonication or stored under – 80 ºC for future use.

Lysates were centrifuged in an Oak Ridge tube at 14,000 rpm for 30 minutes (4 ºC) to remove cell debris. Each supernatant was passed through a 0.45-µm filter prior to application to a 5-mL column of nickel-NTA resin, which was then equlibrated with at least two column volumes of the Lysis Buffer, followed by washings with 2 column volumes (CVs) of 90:10 Lysis Buffer:Elution Buffer (50 mM sodium phosphate (pH 8.0), 300 mM NaCl, 500 mM imidazole, and 5% (v/v) glycerol,

10 mM β-mercaptoethanol). Elution of resin-bound protein was achieved by a stepwise increase to 40% of the Elution buffer (60:40 Lysis Buffer:Elution Buffer) and was indicated by increasing absorbance at 280 nm in the eluted fractions. The presence of 3CL-PR in the eluate was confirmed by the observation of a 34k-Da band upon analysis of fractions by SDS-PAGE (**Figs. S13-S15**), prior to pooling of eluates and dialysis overnight in the Dialysis Buffer (50 mM sodium phosphate (pH 8.0), 300 mM NaCl, 5% (v/v) glycerol, and 10 mM β-mercaptoethanol) at 4 ºC. The cleavage of the N-terminal His-SUMO tag was initiated by adding 0.5 mg His_6-Ulp1 protease into the pooled eluates containing enzyme. The cleaved His-SUMO tag, Ulp1, and any other impurities from the SARS-CoV-2 3CL-PR was trapped in a second HisTrap Ni column. After Ulp1 protease-catalyzed cleavage (**Fig. S15**), the activity of the 3CL-PR was confirmed with an activity assay (data not shown). The concentration of the flowthrough from the second column containing pure SARS-CoV-2 3CL-PR was determined by recording the absorbance at 280 nm on a NanoDrop device and calculated with an extinction coefficient (32,890 $M^{-1}cm^{-1}$) and stored in the Dialysis Buffer at 4 °C. The overall yield of SARS-CoV-2 3CL-PR purification ranged from 25-50 mg protein per liter bacterial culture for Cnstr3 and Cnstr4.

Expression and purification of His_6-HRV-3C protease.

Cleavage of the His_6-tag from His_6-SARS-CoV-2 3CL-PR was carried out using commercial Precise His_6-HRV-3C protease (ThermoFisher) at a ratio of 3.5 units of HRV-3C protease added per mg of His_6-SARS-CoV-2 3CL-PR. For large-scale purification, the cleavage of His_6-SARS-CoV-2 3CL-PR was carried out using His_6-HRV-3C protease to maximize cleavage efficiency while maintaining low cost. Briefly, the plasmid construct for protein expression consisted of a His_6-tag at the N-terminus of the protease coding region, followed by the full sequence of HRV-3C protease. A glycerol stock of *E.coli* containing this plasmid was used to inoculate 10-mL LB

media containing 100 µg/mL carbenicillin, followed by incubation at 37 ºC overnight. The starter culture was then transferred into 1 L of LB media containing 100 µg/mL carbenicillin for large-scale growth. The culture was chilled on ice after OD_{600} reached 0.6-0.8, after which 1 mM IPTG was added to induce protein expression at 18 ºC overnight. Centrifugation was performed at 6,300 x g, 4 ºC for 45-60 minutes to spin down *E.coli* cell pellets for either immediate lysis or storage under – 20 ºC for future use. Resuspension and lysis of the cell pellets was carried out at the same condition as that of SARS-CoV-2 3CL-PR, with the addition of 1 mM $MgSO_4$ and 25 µg/mL DNase to reduce the viscosity of the solution for efficient lysis using a French press. After cell disruption using a French press, the lysate was purified and dialyzed with the same procedure as that used for purification of SARS-CoV-2 3CL-PR. Material that precipitated during dialysis was removed by centrifuging the sample at 4 ºC, 26,000 x g for 45 minutes. The presence of His6-HRV-3C protease in the supernatant was confirmed by the presence of a thick 21-kDa band on SDS-PAGE. The supernatant was collected and concentrated to ~ 5 mg/mL as determined by absorbance measurement using a Nanodrop cell (Biotek Synergy plate reader). The concentrated protein sample was aliquoted into 0.5-mL samples in Eppendorf centrifuge tubes, and stored at -80 ºC for future use. During lysis of bacteria using a French press, the viscosity of the sample led to discernible protein precipitation. DNase and $MgCl_2$ were added with stirring, and the viscosity of the *E.coli* suspension dropped significantly within 30 minutes of stirring. During a second cell disruption attempt using the French press lysis, no protein precipitation was observed, and the protein yield was higher than expected. Figure **S14** shows the SDS-PAGE result of different fractions in HRV 3C protease purification. A band at ~ 21 kDa in the cell lysate and in the eluate from the HisTrap column indicated the the existence of the protein (chromeextension://efaidnbmnnnibpcajpcglclefindmkaj/https://structbio.vanderbilt.edu/wetlab/pr

ivate/vectors/3C/Purif.H6H3C.v6.pdf), which is seen throughout all the samples. Most of the irrelevant proteins were eliminated during the washing step, but the existence of other protein bands suggested that there was still non-specific binding to the affinity column. The overall protein yield was high (app. 48 mg per Liter x 12 Liters= 576 mg protein), which could explain the observed precipitation of protein during dialysis to remove excess imidazole in the elution buffer. The precipitant could not be re-solubilized by application of the HisTrap elution buffer, and purificiation analysis by SDS-PAGE suggested that the precipitated protein also contained HRV 3C protease, but the amount of HRV 3C protease lost in the precipitant was not significant as the final yield of protein remained at 21 mg per liter of *E.coli* culture.

REFERENCES

1. Mellott DM, Tseng CT, Drelich A, Fajtová P, Chenna BC, Kostomiris DH, Hsu J, Zhu J, Taylor ZW, Tat V, Katzfuss A, Li L, Giardini MA, Skinner D, Hirata K, Beck S, Carlin AF, Clark AE, Beretta L, Maneval D, Frueh F, Hurst BL, Wang H, Kocurek KI, Raushel FM, O'Donoghue AJ, de Siqueira-Neto JL, Meek TD, McKerrow JH. A cysteine protease inhibitor blocks SARS-CoV-2 infection of human and monkey cells. ACS Chem Biol. 2021 Mar 31;: PMID: 33140046; PMCID: PMC7605553.

2. Manual, Center for Structural Biology, Vanderbilt University.

3. Palomo JM. Solid-phase peptide synthesis: an overview focused on the preparation of biologically relevant peptides. Rsc Advances. 2014;4(62):32658-72.

ABBREVIATIONS

3CL-PR, 3-chymotrypsin-like protease (or Main protease); Abz, 2-aminobenzoic acid; AMC, 7-amino-4-methylcoumarin; COMU, 1-Cyano-2-ethoxy-2-oxoethylidenaminooxy)dimethylamino-morpholino-carbenium hexafluorophosphate; Dabcyl, 4-[4-(Dimethylamino)phenylazo]benzoyl; DEPC, diethylpyrocarbonate; DIPEA, di-isopropylethylamine; Dnp, di-nitrophenol; DMF, dimethyl formamide; DCM, dichloromethane; DMF, dimethylformamide; DTNB, 5,5'-dithio-bis-(2-nitrobenzoic acid); DTT, dithiothreitol; Edans, (5-((2-aminoethyl)amino)naphthalene-1-sulfonyl; EDTA, ethylenediaminetetraacetic acid; GST, glutathione transferase; HEPES, 4-(2-hydroxyethyl)-1-piperazineethanesulfonic acid; His$_6$, hexa-histidine; human rhinovirus 3CL protease, HRV-3CL; IAM< iodoacetamide; IPTG, isopropyl β-D-1-thiogalactopyranoside; MES, 2-(N-morpholino)ethanesulfonic acid; NEM, N-ethylmaleimide; RFUs, relative fluorescence units; TAPSO, N-[tris(hydroxymethyl)methyl]-3-amino-2-hydroxypropanesulfonic acid; TCEP, triscarboxyethylphosphene; TEA, triethanolamine; and TFA, trifluoroacetic acid.
